# Probing contacts of inhibitor locked in transition states in the catalytic triad of DENV2 type serine protease and its mutants by 1H, 19F and 15 N NMR spectroscopy

**DOI:** 10.1186/s12860-020-00283-0

**Published:** 2020-05-25

**Authors:** Peter Agback, Esmeralda Woestenenk, Tatiana Agback

**Affiliations:** 1 Department of Molecular Sciences, Swedish University of Agricultural Sciences, PO Box 7015, SE-750 07 Uppsala, Sweden; 2 Protein Expression and Characterization Drug Discovery and Development Platform, Science for Life Laboratory, Solna, Sweden

**Keywords:** Dengue, Serine protease, Catalytic triad, NMR, 19F, ligand interaction

## Abstract

**Background:**

Detailed structural knowledge of enzyme-inhibitor complexes trapped in intermediate state is the key for a fundamental understanding of reaction mechanisms taking place in enzymes and is indispensable as a structure-guided drug design tool. Solution state NMR uniquely allows the study of active sites of enzymes in equilibrium between different tautomeric forms. In this study 1H, 19F and 15 N NMR spectroscopy has been used to probe the interaction contacts of inhibitors locked in transition states of the catalytic triad of a serine protease. It was demonstrated on the serotype II Dengue virus NS2B:NS3pro serine protease and its mutants, H51N and S135A, in complex with high-affinity ligands containing trifluoromethyl ketone (tfk) and boronic groups in the C-terminal of tetra-peptides.

**Results:**

Monitoring 19F resonances, shows that only one of the two isomers of the tfk tetra-peptide binds with NS2B:NS3pro and that access to the bulk of the active site is limited. Moreover, there were no bound water found in proximity of the active site for any of the ligands manifesting in a favorable condition for formation of low barrier hydrogen bonds (LBHB) in the catalytic triad. Based on this data we were able to identify a locked conformation of the protein active site. The data also indicates that the different parts of the binding site most likely act independently of each other.

**Conclusions:**

Our reported findings increases the knowledge of the detailed function of the catalytic triad in serine proteases and could facilitate the development of rational structure based inhibitors that can selectively target the NS3 protease of Dengue type II (DENV2) virus. In addition the results shows the usefulness of probing active sites using ^19^F NMR spectroscopy.

## Background

Dengue virus (DENV), with its four common serotypes (DENV 1–4), transmitted predominantly in tropical and subtropical regions by the mosquito *Aedes aegypti*, is currently increasing worldwide, infecting millions of people and causing dengue fever, dengue hemorrhagic fever, and dengue shock syndrome [1]. It belongs to the flavivirus genus, which also includes Zika virus [2, 3], West Nile virus (WNV) [4], and Yellow Fever virus (YFV) [5]. The flaviviral RNA genome consists of one open chain, encoding a single polyprotein including three structural proteins (C, prM, and E) and seven nonstructural proteins (NS1, NS2A, NS2B, NS3, NS4A, NS4B, and NS5) [6]. It was originally believed that the NS3 domain encoded the functional protease. However later studies showed that the protease is a two component system [7]. In this dimeric protease the virally encoded serine protease lies in the N-terminal protease domain of NS3 (NS3pro), with NS2B serving as a cofactor. A segment of minimally 40 residues (amino acids 1394–1440 of the polyprotein) suffices for full proteolytic activity of NS3pro [8]. The NS2B:NS3pro serine proteases have been studied intensively due to their critical role in polyprotein maturation and viral infectivity [9].

The complexity of the ‘open’ and ‘closed’ conformations of DENV protease and the significance of a covalent linker between NS2B and NS3 for enzyme activity and structure has been a topic of much discussion in recent years, and was recently summarized by Hill et al. [9]. Their findings suggest that unlinked constructs are better suited for future drug development efforts. We recently assigned unlinked DENV2 NS2B:NS3pro in complex with boronic acid inhibitor (I) where all key amino acids in the catalytic triad and oxyanion hole were successfully identified (BMRB 26996), [10]. This was lacking in the earlier reported NMR assignments performed on the linked construct.

A common feature of serine proteases is the His-Asp-Ser catalytic triad; for DENV NS3pro the catalytic residues are H51, D75 and S135. The exact nature of the hydrogen bonds in the catalytic triad is of importance in order to understand the mechanism. It has been suggested that the aspartate hydrogen bond to the histidine is a delocalized low barrier hydrogen bond (LBHB) [11, 12]. LBHB is thus important to the understanding of the structure of the tetrahedral transition state for the functioning enzyme. Other parts involved in the protease function are: the peptide binding site and the oxyanion hole which stabilizes the negative charge on a deprotonated oxygen [13]. There are several reports on serine proteases where the interaction between the catalytic triad and boronic or aldehyde substrate analogue inhibitors have been studied and there is an understanding that in different type of serine proteases different mechanisms could prevail involving different modes of inhibitor binding [12–18]. Remarkably, in the x-ray structure of West Nile virus (WNV) NS2B:NS3pro with a short peptide boronic acid type of inhibitor recently obtained the inhibitor orientation in the active site is complemented by interaction with an additional molecule, glycerol, present in the enzyme [19]. This indicates that catalytic active site in serine proteases could exhibit high plasticity in an apparent mobile environment.

In our earlier NMR studies we demonstrated the existence of the above mentioned LBHB between H51 and D75 of the catalytic triad in the transition state of DENV2 NS2B:NS3pro with substrate-analogue boronic acid inhibitor Bz-Nle-Lys-Arg-Arg-B(OH)2, (compound I, Table 1) [20]. To our knowledge, this is the first time the existence of a LBHB type complex in serine proteases, as has been predicted [12], could be demonstrated by NMR spectroscopy in a biological system. The unusual large low-filed shift of N^δ1^H (19.93 ppm) of H51 combined with a N-H splitting of only 52 Hz clearly indicated the presence of LBHB [20].

**Table 1:**
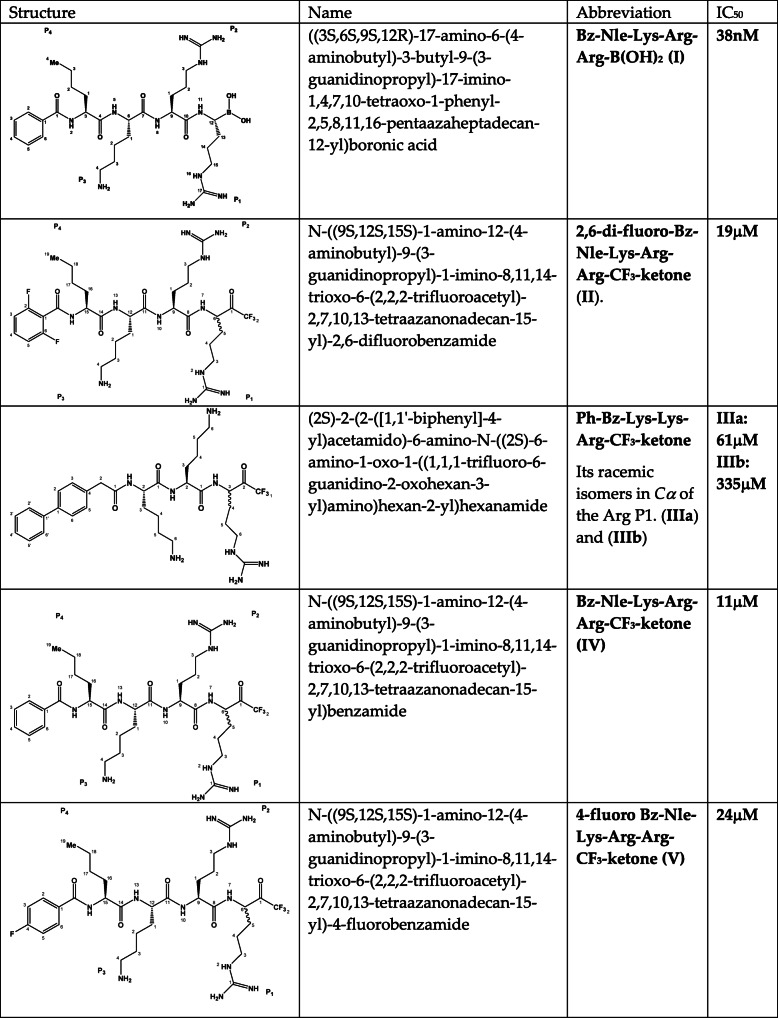
Structures and abbreviations of the compounds used in this study

There are several recent papers where the conformation of inhibited dengue protease is studied by NMR spectroscopy [21–24]. Despite this recent progress there are unanswered questions related to the conformation of the inhibitor-trapped catalytic triad and the role of NS2B in catalysis. In this study we focused primary on the first of these questions. We used ^1^H, ^19^F and ^15^N NMR spectroscopy to study an unlinked construct of DENV2 NS2B:NS3pro in complex with two different inhibitor types presumably mimicking the intermediate stage of substrate binding: a boronic acid (I) and a set of trifluoromethyl keton (tfk) inhibitors (II)-(V) (Table 1). The analysis of the chemical shift perturbations (CSP) differences of the backbone of the parent NS2B:NS3pro protein and its catalytic mutants, S135A and H51N, induced by boronic (I) and tfk (II)-(V) inhibitors, allow us to study conformational changes in the active site. We believe that a better understanding of the atomic interactions in the active site will ultimately lead to improved NS3 protease-targeting drugs.

## Results

### Complexes of the dengue II NS2B:NS3pro protein with peptide type -CF_3_-ketone inhibitors

#### Monitoring of the binding ability of the peptide type -CF_3_-ketone isomers to the NS2B:NS3pro by ^19^F NMR

**Fig. 1:**
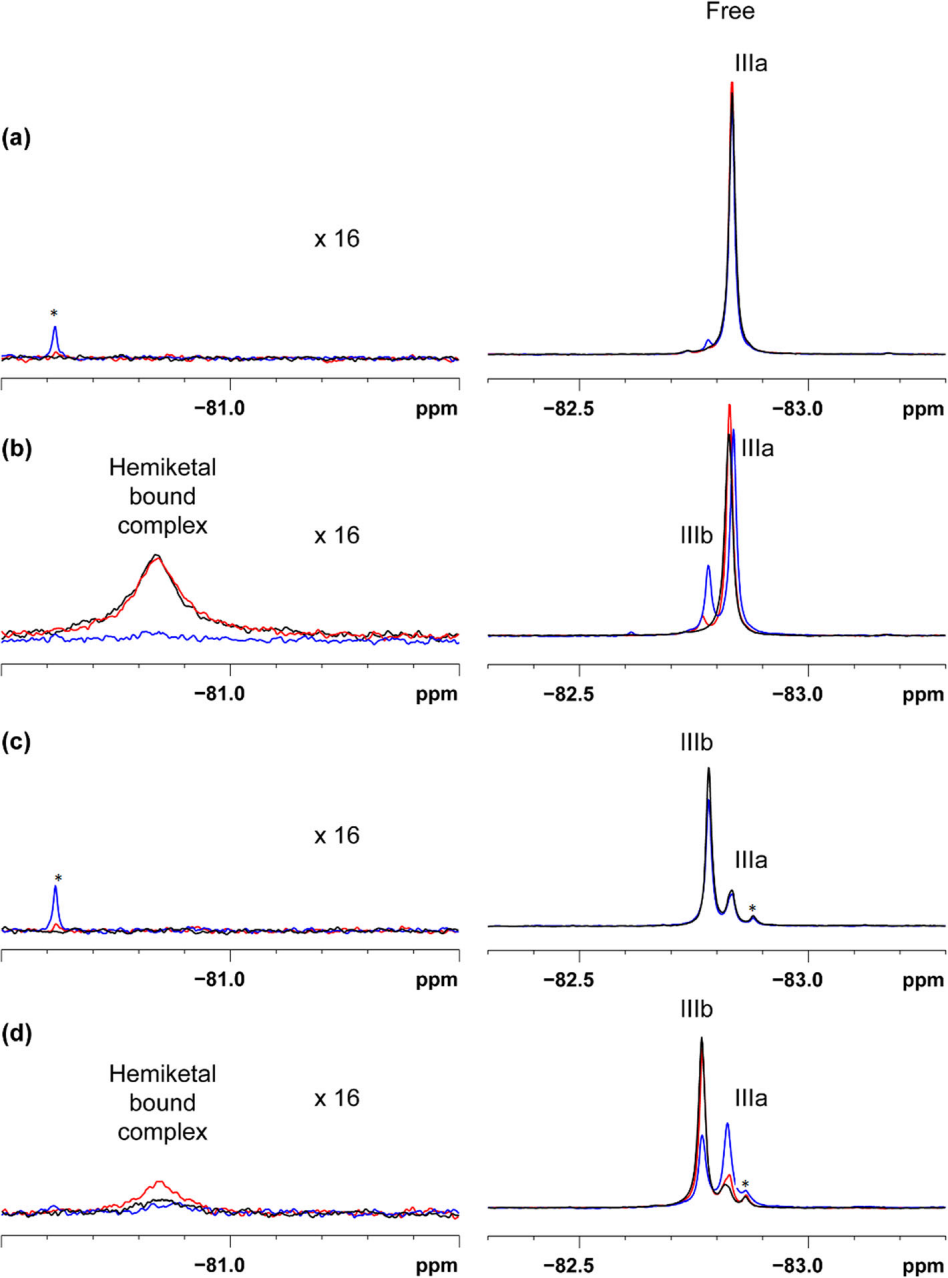
^**19**^**F spectra for CF**_**3**_**ketones (IIIa and IIIb) with NS2B:NS3pro.** Superposition of ^19^F spectra between − 80.5 to − 81.5 (intensities scaled up 16 times) and − 82.3 to − 83.3 ppm obtained at 25 °C. Spectra were collected: shortly after sample preparation (black), following incubation at 25 °C for 12 days (red) and for two months (blue). The signals of the free isomers of the inhibitors at − 82.83 ppm and – 82.78 ppm are assigned to (**IIIa**) and (**IIIb**), correspondingly. Impurity signals are marked with *

The subside part of peptide types of inhibitors either improves or prevents correct binding with the enzyme in the most favourable way [16]. One way to probe stereo-selectivity in enzymes is to study an inhibitor with the wrong stereochemistry around the carbon, Cα, of the P1 (Arg) (for abbreviation see Table 1). The key question is: could both isomers bind to NS2B:NS3pro complex? If yes, is there any difference in affinity between them. To challenge these questions two separated diastereomers of Ph-Bz-Lys-Lys-Arg-CF3-ketone (III) (Table 1) with low affinity were used to observe the binding. The isolated diastereomers were kept in solution with and without the NS2B/NS3 complex for two months with measuring at several points. For the first separated isomer (IIIa), only one ^19^F resonance is observed at − 82.83 ppm in the unbound state (Fig. 1a). After two months some small amount of isomer (IIIb) can be seen to appear. With the addition of protein there are two signals (Fig. 1b): the downfield broad signal at − 80.84 ppm belonging to the ligand in the bound state and the more upfield signal at − 82.83 ppm belonging to the unbound inhibitor. For the second isomer (IIIb) the unbound signal is located at − 82.78 ppm (Fig. 1c), note that it is not 100% pure some amount of isomer (IIIa) can be seen. With protein addition, the signal of the bound complex at − 80.85 ppm is observed only at the noise level (Fig. 1d), which indicates that affinity is very low. After 2 months incubation of the samples at 25 °C (blue line Fig. 1b, d) the fraction in bound state of the complex is not observed, instead two signals of unbound ligand at − 82.78 and – 82.83 ppm belonging to the isomers (IIIb) and (IIIa) respectively. The epimerisation is fully complete for isomer (IIIb) compared to isomer (IIIa) (Fig. 1d). In the spectra without protein the signals showed only a minor change in intensity due to epimerization (Fig. 1a, c).

#### NS2B:NS3pro binds to one isomer of the racemic mixture of Bz-Nle-Lys-Arg-Arg-CF_3_-ketone (II)

A racemic mixture of Cα of the P1 (Arg) in the free state of (IV) was manifested by the presence of two sets of signals in the ^19^F spectrum: at − 82.75 ppm (isomer IVb) and − 82.82 ppm (isomer IVa). In accordance with the data presented above for tfk III, for tfk type IV isomer IVb was expected to have lower affinity than IVa. Indeed, by replacing tfk (IV) in complex with NS2B:NS3pro with the more potent boronic (I) inhibitor, the signal of only one isomer (IVa) re-appeared (Fig. S1). It was concluded, firstly, that for all tfk type inhibitor (II), (IV) and (V) the high affinity isomer has more upfield ^19^F signals. Secondly, that NS2B:NS3pro binds only one of the tfk isomers.

### Limited access of bulk and bound water to the active site of the complex of NS2B:NS3pro with boronic (I) and tfk (II) inhibitors

#### Probing water accessibility of the NS2B:NS3pro complex with tfk inhibitor by ^19^F NMR

The accessibility of water in the binding site of the complex with bound inhibitor was tested by studying the ^19^F spectra of the complexes NS2B:NS3pro (II) in two different solvents: H_2_O and D_2_O (Fig. S2). No shift of the ^19^F resonance at − 81.36 ppm belonging to the –CF_3_ group of the bound inhibitor (II) was observed in contrast to the signal in form the free inhibitor which moved up field by ca 33 Hz (Fig. S2b). This data indicates that the warhead of the ligand is deeply buried in the body of the protein complex and that there is limited access of bulk water in the active site. Noteworthy, that for ^19^F of the benzene ring signals of the bound complex located at − 114.57 ppm an upfield shift of ca 38 Hz was observed. This CSP was scaled versus the signals of the same group of the unbound ligand, ca 48 Hz (Fig. S2c). The conclusion is that ^19^F nuclei of the benzene ring of the bound inhibitor are partly exposed to the solvent even in the bound state.

#### Bound water in the complex of NS2B:NS3pro with inhibitors (I) and (II)

To identify the presence of bound water in the NS2B:NS3pro ligand complexes we used an approach developed by us earlier in which one can differentiate between bulk water and more tightly bound water using [25]. In the current study the advantages of the unlinked NS2B:NS3pro construct was exploited, allowing separate detection of NH contacts with closely located bound water either for NS2B or NS3pro.

The superposition of the 2D plane ^1^H-^15^N of the 3D experiment for water detection of NS2B:NS3pro complexed with (I) and (II) are presented in Fig. S3. For both inhibitors (I) and (II) very similar water interaction patterns can be observed (Fig. S3). Indeed, the cross peaks for amide protons of NS3pro with largest intensity attributed to the contacts with bound water were observed in both inhibitor complexes: K15, W89; R107, G121, S127, S131, N152, G153, A166, G159, V169, (Fig. S3a and b). Many cross peaks showing amide protons involved in exchange with bulk water are in the crowded center of the spectrum but some are clearly observed outside this area: Y33, I36, G32, S68, G103, N105, T122, D129 and I182. A few bound water contacts with amide protons in the complex NS2B:NS3pro with (I) were registered for NS2B: S70, I73, D81 and M84 (Fig. S3a). The corresponding cross peaks of amide protons involved in exchange with bulk water are: R55, A65, G69, S71 and E80.

Significantly all amide protons involved in detected contacts either with bound or bulk waters are away from the catalytic triad. These data lead us to two conclusions. Firstly, despite the difference in the warheads which thus have different type of interactions of inhibitors (I) and (II) in the catalytic triad, the similarity in bound waters suggests only minor implications on the overall architecture of the water pattern. Secondly, not observing either bound or bulk water in proximity to the active site of NS2B:NS3pro indicates that the conditions in the tetrahedral intermediates are hydrophobic and are optimal for creation of LBHB bonds in the catalytic triad [11].

### Interaction in the oxyanion hole of NS2B:NS3pro bound to boronic (I) and tfk (II) inhibitors

The active site of serine proteases consists of a catalytic triad (S135-H51-D75 for DENV2 NS3), but also an oxyanion hole. It has been discussed that the oxyanion hole plays an important role in stabilizing the tetrahedral intermediate during the attack by a substrate in the active site of the enzyme forming a Michaelis complex [13]. The effective interaction of the substrate-based tetra-peptide inhibitors with oxyanion hole varies depending on the type of warhead [12, 16].

To identify the interactions of inhibitors (**I**) and (**II**) in the oxyanion hole, a reliable assignment of the amide group resonances belonging to the active site of the NS3pro should be achieved. We had observed that the apo protein was prone to degradation, which made assignment challenging. Instead, we compared data sets available in the public domain with assignment of the DENV2 NS2B:NS3pro apo form [26], complexes with small molecules [27], tfk type of inhibitor (deposited to BMRB id 19,305) [21], *complex with aprotinin* (deposited to BMRB id 18,266) [28], and with boronic type of inhibitors [29], and by us (deposited to BMRB id 26,996), [10]. For the catalytic triad, the assignments of H51 and D75 are corroborated for all data sets. The differences between the data sets are mainly related to the fragment of NS3pro sequence between two prolines P132-G133-T134-S135-G136-S137-P138 forming the oxyanion hole. These observed discrepancies are possibly due to differences in interaction between different type of ligands and active sites. In some cases the resonances were not assigned.

In our earlier study we have unambiguously assigned the resonances of amide groups belonging to the S137, G136, S135, T134 and G133 residues of the NS3pro in complex with tetra peptide boronic acid inhibitor (**I**) [10]. Unfortunately it was not possible to compare our assignment with the closest analogue, the dipeptide boronic acid inhibitor, due to the incomplete assignment [29]. Comparison of the amide chemical shift of the oxyanion hole between complex and apo form shows that CSP induced by the boronic acid is not large (ca 0.3 ppm). This is much less than expected if an OH group/or groups of the boronic tetrahedral intermediate is involved in hydrogen bonding with NH of the oxyanion hole or other direct contacts.

**Fig. 2:**
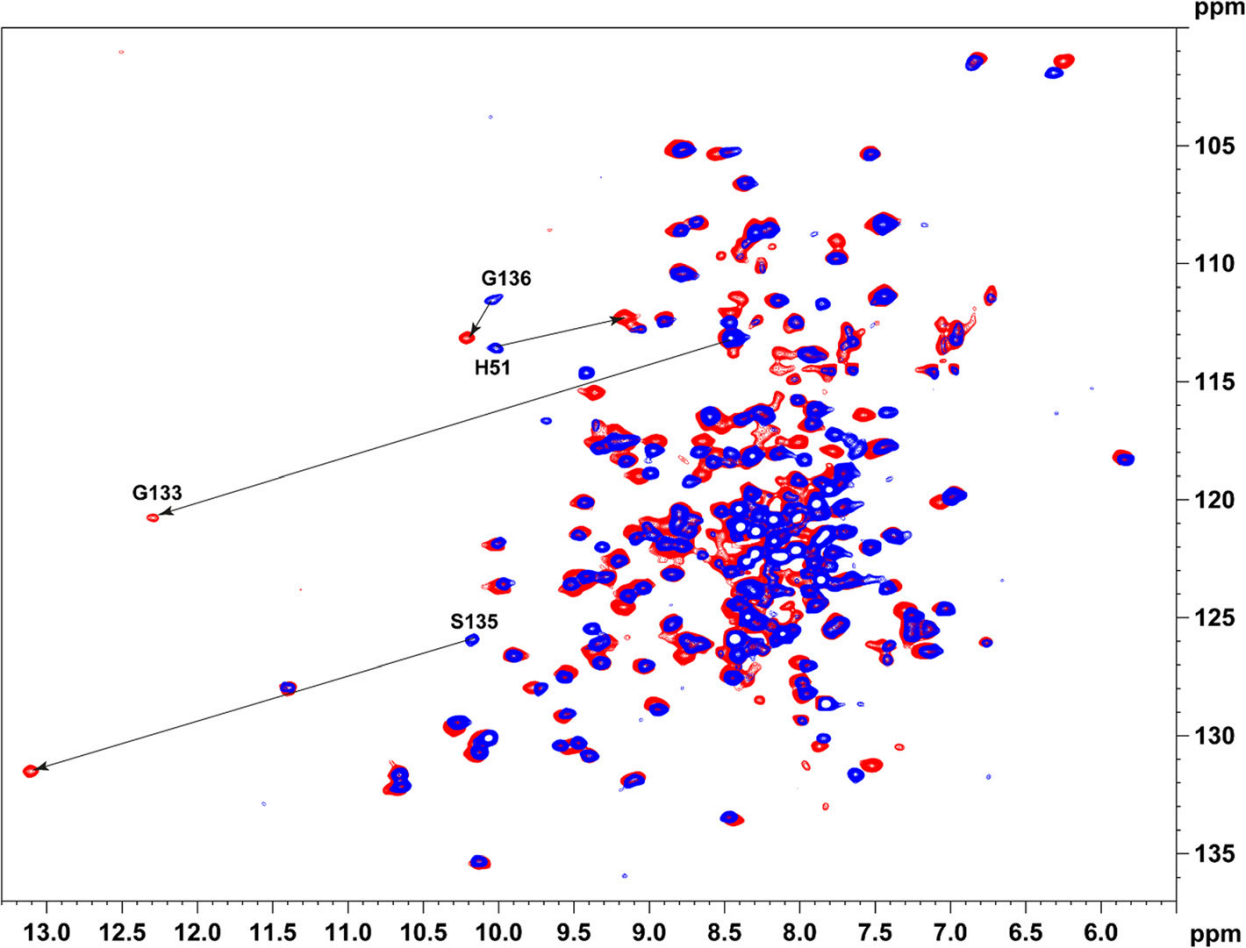
^**1**^**H-**^**15**^**N TROSY spectra of**^**15**^**N labeled NS3pro, unlabeled NS2B with inhibitors (I, II).** Superposition of the ^1^H-^15^N TROSY spectra of ^15^N labeled NS3pro, unlabeled NS2B with boronic acid (**I**) and tfk (**II**) inhibitors are shown in blue and red, respectively. The cross peaks of amino acids with the largest CSP are connected by arrows and marked according to the residue number in sequence

In contrast, in the complex formed with tfk type inhibitor (**II**), a strong down field shift of amide resonances S135 and G133 are detected (for ^1^H ca 3 and 4 ppm respectively; Fig. 2 and Fig. S4). This is in good agreement with HCV NS4A:NS3 complexes earlier reported where the hemi-ketal oxygen of the tetrahedral intermediate is involved in a hydrogen bond with the NH of oxyanion hole amides [30].

It is expected that the D75 side chain can form hydrogen bonds not only with the Hδ1 side chain hydrogen of H51 but also with its backbone hydrogen. Indeed it was shown for HCV NS4A:NS3 [31] that a H51-HN chemical-shift of ca 10.6 ppm was observed which is consistent with the formation of a H51-HN….D81-Oδ hydrogen bond seen in crystal structures (numbering given in [31]). In the apo DENV2 NS2B:NS3pro the H51-HN is observed as a double peak (ca 10.56/114.44 and 10.18/114.37 ppm). In the tfk (**II**) complex the ^1^H/^15^N of H51-HN moves up-field (9.17/112.28 ppm), while in the boronic acid (**I**) complex it moves up-field but to a lesser degree (10.03/113.50 ppm; Fig. S5) indicating that hydrogen bonding between H51-HN and D75-Oδ is weakened in both cases but to different extent. In an X-ray structure obtained for WNV NS2B-NS3pro with a boronic dipeptidic inhibitor the distance between the corresponding positions is 2.67 Å, indicating the possibility to form a hydrogen bond [19].

### Low field ^1^H of NS2B:NS3pro complexed with boronic acid (I) and tfk (II) inhibitors

Adding **(I)** and **(II)** inhibitor to NS2B:NS3pro leads to a significant change in the ^1^H low field part of the spectrum between 20 and 13 ppm compared to the apo form where no resonances were observed in this region.

**Fig. 3:**
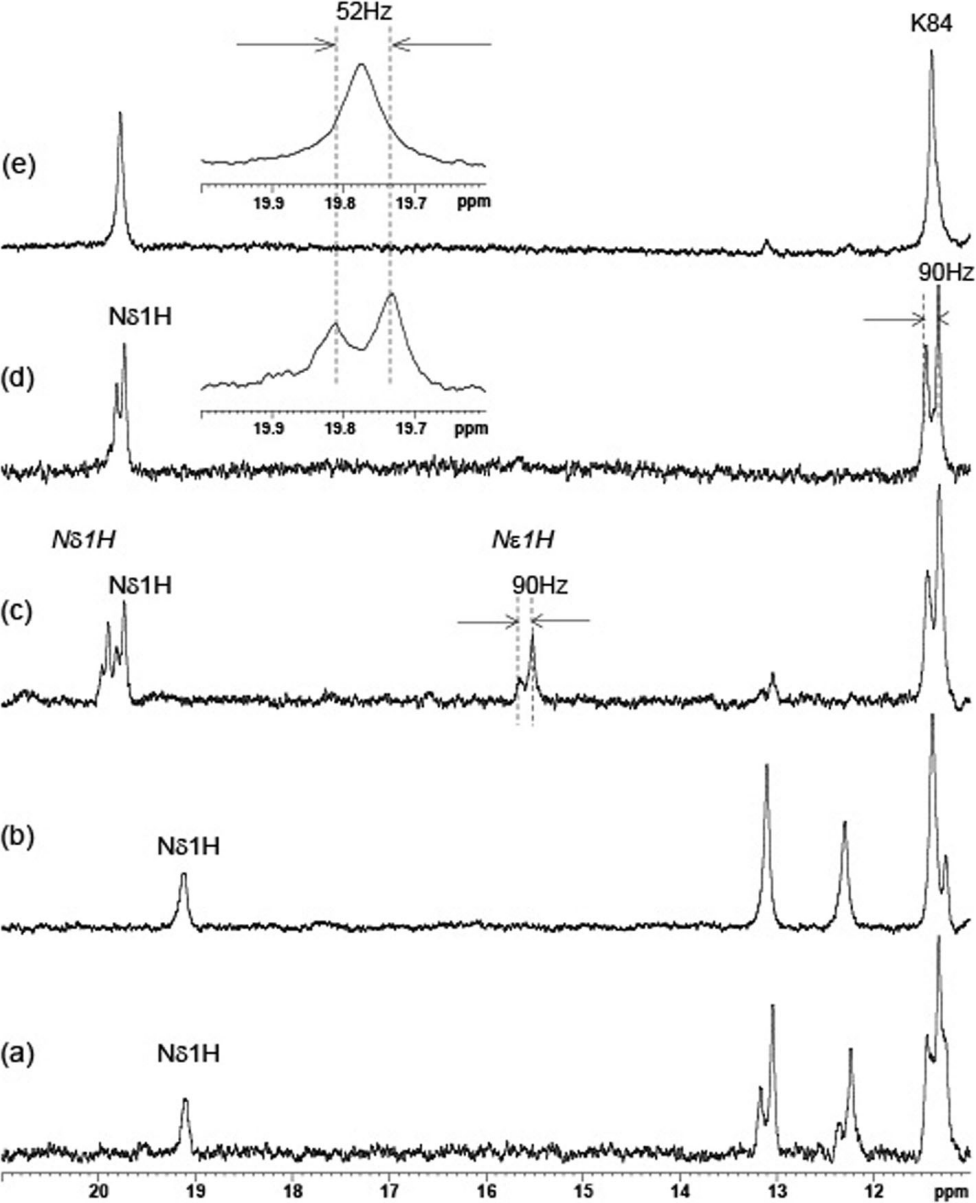
**1D**^**1**^**H spectra of the region 11-21 ppm of inhibitors (I, II) with**^**15**^**N**^**13**^**C NS2B:NS3pro.** 1D ^1^H spectra of the region 11-21 ppm of: (a) ^15^N^13^C-NS2B: ^15^N^13^C-NS3pro with 2,6-di-fluoro-Bz-Nle-Lys-Arg-Arg-CF_3_-ketone (**II**) in pH 6.0 MES buffer, (b) ^15^N^13^C-NS2B:NS3pro with 2,6-di-fluoro-Bz-Nle-Lys-Arg-Arg-CF_3_-ketone (**II**) in pH 6.0 MES buffer, (c) ^15^N^13^C-NS2B: ^15^N^13^C-NS3pro with Bz-Nle-Lys-Arg-Arg-B(OH)_2_ (**I**) in pH 6.0 MES buffer, (d)) ^15^N^13^C-NS2B: ^15^N^13^C-NS3pro with Bz-Nle-Lys-Arg-Arg-B(OH)_2_ (**I**) in pH 8.5 Tris buffer and (e) ^15^N^13^C-NS2B:NS3pro with Bz-Nle-Lys-Arg-Arg-B(OH)_2_ (**I**) in pH 8.5 Tris buffer. The small inserts in (d) and (e) show N^δ1^H of H51 second tautomer (in **bold**) at 19.77 ppm with the one bond J-coupling: ^1^J_Nδ1H_ = 52+/− 2 Hz. For first tautomer (c in *italic*) ^1^J_Nδ1H_ = 52+/− 2 Hz for 19.93 ppm of N^δ1^H of H51 and ^1^J_Nε2H_ = 90+/− 2 Hz for 15.59 ppm of N^ε2^H of H51. As reference amide backbone ^1^J_NH_ = 90+/− 2 Hz of the amino acid K84 at 11.39 ppm (d) is shown

The presence in extreme low field of a singlet signal at 19.11 ppm of ^15^N^13^C-labeled NS2B:NS3pro with **(II)** (Fig. 3a and b) is well in agreement with other NMR studies of proteases in complex with a similar type of tfk inhibitor [11, 32–35]. Its line width is ca 55 Hz which is broader than the line widths for another imide protons (ca 35 Hz) and it does not change when unlabeled NS3pro is used (Fig. 3b). Noteworthy, we also fail to observe one bond splitting with ^15^N nuclei and any correlation in ^1^H-^15^N TROSY spectrum even at lower temp (5^ο^C) (data not shown). The chemical shift of this resonance is pH independent in the range between 5.5 and 8.5 which corroborates earlier reports for chymotrypsin protease [32]. Indeed, for a non-hydrogen bonded histidine, when participating in complex formation it would be expected to show chemical shift dependence on pH whereas histidine bound in a complex would not [36–38]. But there is a difference. Indeed the intensity of the signal for the *N*-Ac**F**-CH3 complex reported by Cassidy et al. persists at pH as high as 10 and then falls off sharply [32]. But for NS2B:NS3pro bound to **(II)** the intensity depends on pH with the largest peak observed at pH 6.0 (Fig. S6).

Low field ^1^H NMR spectra of ^15^N^13^C-labeled NS2B:NS3pro with inhibitor (**I**) (Fig. 3c-e) are different compared to the spectrum of the complex inhibitor **II** (Fig. 3a, b). As has been reported earlier by us there are four signals observed between 20.1–19.6 ppm and two signals around 15.59 ppm (Fig. 3c) of NS2B:NS3pro with **I** at pH = 6.0 and one singlet for complex with **II** [20]. When NS3pro is unlabeled (Fig. 3e) or when decoupling of the ^15^N nuclei is applied (data not shown) the resonances are reduced to two signals at 19.93 ppm and 19.77 ppm, and one signal at 15.57 ppm. The J_NH_ couplings are 52 Hz, 52 Hz and 90 Hz correspondingly (Fig. 3 c, d). None of the signals in this region shift between pH 5.5 and 8.5 on their chemical shift, so H51 remains fully protonated and H-bonded over the investigated pH range. The most intense signal at 19.77 (Fig. 3d) is the most persistent in all spectra at different buffers and pH (Fig. 3 c, d, e). The other two signals at 19.93 and 15.57 are not always observed and depend on sample conditions (e.g. Figure 3d,e Tris buffer pH 8.5). The signals at 19.93 and 19.77 ppm were attributed to (N)H^δ1^ of His 51 [20]. The proposed assignment corroborates observations in X-ray structures of Dengue type proteases showing a possible hydrogen bonding between N^δ1^ of a histidine and aspartic acid. Further proof that those signals belong to the H51 in the catalytic triad is that in the spectra of the H51N mutant in complex with (**II**) inhibitors there were no low field signals similar to 19.93, 19.77 and 15.57 ppm.

### Characterization of S135A and H51N mutants

#### Peptide boronic acid (I) and tfk (II) inhibitors do not interact with the S135A mutant

To understand the role of the catalytic S135 in the interaction with the boronic and tfk inhibitors, ^15^N^13^C-labeled NS2B:NS3pro (S135A) mutant was produced. The ^1^H-^15^N TROSY spectrum of the apo form of the S135A mutant is very similar to the corresponding spectrum of the wild-type NS2B:NS3pro (Fig. S7). Almost all CSPs detected were below 0.1 ppm. The largest CSPs were observed for H51 and G136 (Fig. S7a). For some cross peaks that are clearly detected as single in the spectrum of the S135A, in the wild-type protein a second cross peak with weaker intensity is present. This is observed for both NS3 and NS2B [in Fig. S7 boxes (a) and (b) a few of those cross peaks are marked by arrows]. It is likely that the major form in wild-type protein and the only form in S135A mutant represent a conformation where the catalytic triad is not formed. It is premature to speculate what the minor form stands for.

The ^1^H-^15^N TROSY spectra of the S135A apo form and those of the S135A mutant complexes with inhibitors (**I**) and (**II**) are almost identical (Fig. S8a). This result was confirmed using ^19^F nuclei as a probe to monitor any interaction between ^15^N^13^C-NS2B:NS3pro (S135A) and **(II)**, followed by the addition of **(I)**: there are no broad signals in regions − 81.0 to − 83.5 ppm and − 114 to − 116.5 ppm which can be attributed to the bound complex (Fig. S8b), i.e. no binding was observed. Similar results were reported for interactions of small non-peptide inhibitors with NS2B:NS3pro (S135A) [27].

#### Interaction of the H51N mutant with boronic acid (I) and tfk (II) inhibitors

Another mutation introduced in the catalytic triad D75-H51-S135 was H51N. The superposition of the ^1^H-^15^N TROSY spectra of the apo form of NS2B:NS3pro (H51N) and wild type NS2B:NS3pro reveals several clear CSPs (between 0.1–0.3 ppm; Fig. S9a), in contrast to the findings for the S135A mutant. For NS3pro, the largest CSPs belonging to the unambiguously assigned and non-overlapping area of the spectrum were observed for amino acids close to N51: V52 (248 Hz), T53 (168 Hz), R54 (44 Hz), G55 (54 Hz) and G44 (84 Hz). Some NS2B amino acids had CSPs, but they are all below 0.1 ppm. Comparison of the spectra of the apo form of mutants H51N and S135A (Fig. S9b) shows that the most significant differences are the CSPs of the amino acids V52, T53, T48 and M49 near H51N. The disappearance of K61 and W89 (NS3pro) and R60 and K87 (NS2B) cross peaks in NS2B:NS3pro (H51N) could be due to the presence of slow exchange between different conformations.

H51N mutation reduces the affinity of the tfk inhibitor. This was tested by using ^19^F as a probe to monitor the bound complex formation as mentioned above. The intensity of the signals of the bound complex is decreased compared to those of the wild-type protein (Fig. S10a) even if the same protein:inhibitor ratio was used in all experiments. This observation is supported by the appearance of weak intensity cross peaks in the ^1^H-^15^N TROSY spectra (Fig. S10b and c), indicative of the presence of a small amount of complex of H51N mutant with both inhibitors. Unfortunately, we cannot identify the maximum CSP in the ^1^H-^15^N TROSY spectra of the complexes of both inhibitors with H51N. This is due to insufficient ligand solubility preventing us from reaching saturation of the complex. Additionally, the apo form of the H51N mutant is not stable at 25 °C and degraded during a few hours. The ^19^F signal of the bound complex of the H51N mutant with the 2, 6-di-fluoro-Bz groups of tfk (**II**) is shifted significantly downfield, − 78.10 ppm, compared to corresponding bound complex with wild type protein, − 81.36 ppm (Fig. S10a), indicating that in the former the ^19^F nucleus is more deshielded than in the latter.

## Discussion

We studied two types of inhibitors, boronic acid (**I**) and tfk (**II**) in complex with dengue virus protease. This type of inhibitors have been used by several groups to target serine proteases by mimicking the intermediate complex of the substrate to form a tetrahedral intermediate. Monitoring ^19^F resonances it was demonstrated that only one isomer of tfk type of inhibitors binds with DENV2 NS2B:NS3pro with high affinity. Unfortunately its configuration of C_α_ of the P1 (Arg) is still unknown. The other isomer of tfk inhibitors was undergoing quick epimerization through interaction with the enzyme. The mechanism of epimerization can be explained by enzymatic enolization postulated in aldehydes, ketones and esters and more studies are in progress [39, 40]. For boronic acid (**I**) inhibitor the synthetic root allows to keep the desirable D or L stereochemistry. Noteworthy, the L isomer of boronic acid (**I**) did not bind with DENV2 NS2B:NS3pro (data not shown). In general our data corroborates earlier proposal that the model of binding ability of either L or D isomers of boronic acid (**I**) or tfk (**II**) inhibitors cannot be extended to the family of serine proteases as a whole [16]. Importantly, as predicted the selection of isomers binding in active site of DENV2 NS2B:NS3pro was unique.

We also confirmed that binding inhibitors, boronic acid (**I**) and tfk (**II**), to DENV2 NS2B:NS3pro results in the formation of different complexes with high affinity. ^1^H NMR has been used to observe the N^δ1^H proton shared between H51 and D75 which show the presence of strong hydrogen bonds called low-barrier hydrogen bonds (LBHB) [11]. We have found that for the complex of the boronic acid inhibitor (**I**) with NS2B:NS3pro the one-bond coupling constant N^δ1^H belonging to the catalytic H51 is about 38 Hz less than commonly reported for corresponding coupling in serine proteases which are claimed to be in range 87-95 Hz [41]. Another striking feature is that signals at 19.933 ppm and 19.772 ppm assigned by us to N^δ1^H protons of the H51 in the catalytic triad are observed at almost 1.0 ppm more downfield than so far reported for either N^ε2^H or N^δ1^H protons in protonated His induced by inhibitors [33, 41]. It is noteworthy that the observed chemical shifts and one-bond coupling constants obtained in this study for the complex with boronic acid (**I**) are in accordance with the predicted ones for LBHB type of binding if one extrapolates the results of NMR study of chemical shifts and coupling constants of the histidines within the catalytic domain of the xylanase Cex from *Cellulomonas fimi* [36]. Nevertheless, the result that N^δ1^H protons of the H51 in the catalytic triad of the complexes with (**I**) are more down shifted than for complex with (**II**) is in contradiction with no unusual structural and functional features of boronic acid type inhibitor complexes reported in the studies [42–44]. It also contradicts the latest computational study performed on similar models of inhibitor types (**I)** and (**II)** [12]. One way to rationalize this discrepancy is by differences in the strengthening of the hydrogen bond between H51 and D75 resulting from a decrease in the dielectric constant due to the substrate excluding water from the active site or by the induction of steric compression between H51 and D75 [33, 45, 46]. The accessibility of water to the catalytic triad in complex with bound inhibitor is a key question in order to evaluate the mechanism of catalyst in serine proteases and specifically fulfilling the condition of creation of the LBHB complexes in the triad [11]. In this study we used ^19^F NMR to probe protein and ligand to solvent exposure via solvent isotope shifts [47]. Indeed, it is known that ^19^F resonances are very sensitive to solvents and that the CS of the ^19^F in molecules would differ depending on the degree of exposure to different solvents. We have shown that for both inhibitors (**I**) and (**II**) bound to DENV2 NS2B:NS3pro, bulk water is excluded from the active site which is favorable to the LBHB complex formation. Moreover, it is known that bound water deeply trapped in the interior of protein can play important structural role. To identify the presence of the bound water in the protein - ligand complexes we used an approach earlier developed by us [25]. All of the bound waters found in the complexes are similarly distributed and located outside the active site of the enzyme and are unlikely to play any role in the stabilization of the catalytic triad.

**Fig. 4:**
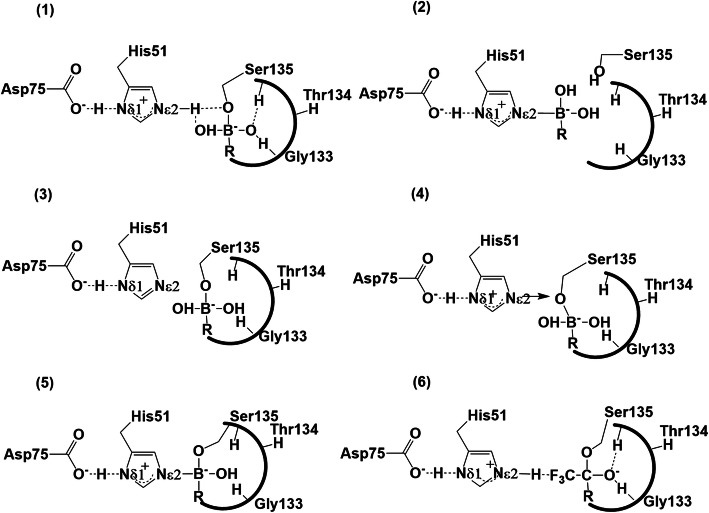
**Schematic representation of possible tautomer forms of the NS2B:NS3pro active site inhibitors (I, II).** Schematic representation of possible tautomer forms of the NS2B:NS3pro active site with boronic acid inhibitor (**I)**: Bz-Nle-Lys-Arg-Arg-B(OH)_2_ [1]– [5] and with tfk inhibitor (**II**): di-fluoro-Bz-Nle-Lys-Arg-Arg-CF_3_-ketone [6]

As a next step we examined the possible different tautomeric structures of the tetrahedral intermediates formed in the catalytic triad and oxyanion hole. They have previously been discussed in the studies of other serine proteases (Figs. 4), [16–18, 37, 42]. To be able to perform this analysis we first need to summarize the results obtained on mutants. In this study two different NS2B:NS3pro catalytic mutants, H51N and S135A, have been used.

In wild type apo NS2B:NS3pro a double set of signals were clearly observed both for NS2B and for NS3pro [10]. For the S135A mutant, amide cross peaks belonging to the catalytic H51 and G136 observed as double peaks in wild type apo are present as single sets of peaks in S135A spectra. They also experienced the largest CSP (Fig. S7). For amino residues located outside the influence of the point mutation, the double set of cross peaks “collapsed” to one set. This suggests that the S135A mutant seems to adopt predominantly one conformation, which also is present in the wild type protein. We argue that two sets of signals observed in apo form of wild type NS2B:NS3pro may not be due to conformation exchange between the open and closed conformation of NS2B rather that they may be due to the presence of an equilibrium between neutral S135 and H51 vs pre-organized complex in the catalytic triad S135-H51-D75. The only proof so far is that in the ^1^H-^15^N spectrum of the apo S135A mutant the cross peaks are almost fully superimposed with one set of signals and the other set is not observed.

In the S135A mutant, the mechanism for the abstraction of the proton from the S135 hydroxyl, which then reacts with the peptide carbonyl of the tfk (**II**) or boronic acid (**I**) inhibitors to form a tetrahedral adduct is obviously omitted due to the absence of S135 hydroxyl. Nevertheless, there is a study where the possibility of a complex with a direct covalently bonded boron nucleus of inhibitor and N^ε2^ of H51 of protease was proposed for a different serine protease, (Fig. 4) [2, 18, 42]. But according to our data on the S135A mutant this type of complex should be ruled out for the DENV2 NS2B:NS3 proteases due to the complete absence of any binding with boronic acid inhibitor (**I**).

Moreover, this experimental data also indicates that even the binding of the P1 and P2 (Table 1) tetra peptide of the inhibitors with corresponding binding pockets, S1 and S2, of NS3pro also did not take place in S135A mutant. This allowed us to conclude that warhead interaction with the catalytic triad is the key step in binding and may possibly lead to allosteric induced transformation of the binding pockets, S1 and S2, to fit to the peptide part of the inhibitors.

The conclusion that the formation of the tetrahedral intermediate is taken place through interaction with S135 agrees with NMR data obtained for catalytic mutant H51N. The binding of tfk inhibitor (**II**) to NS2B:NS3pro_H51N_ was observed, albeit with reduced affinity. We propose that in this mutant the attack on the ketone group of the inhibitor warhead by the OH group of S135 takes place and a covalent adduct can be formed but is not stabilized: even though the leaving proton can be accepted by NH_2_ of N51 there is no possibility of stabilization of the catalytic triad as a whole involving D75. The same argument is valid for the boronic acid inhibitor (**I**) because even though the complex has been observed the equilibrium was shifted to the apo form and saturation of the protease and inhibitor complex could not be reached. We can thus conclude that H51 plays a critically supportive role for the stabilization of the tetrahedral adduct created between the inhibitor warhead and the catalytic triad.

Based on the experimental results presented above for the S135A and H51N mutants we ruled out the possibility of formation of a histidine adduct complex [2] (Fig. 4) described earlier for α-lytic proteases [18, 42], as well as structure [3] which was used in a structure refinement protocol based on NMR nOe data for the complex NS2B:NS3pro with (II) [21]. We arrive at this conclusion based on the result that for both of S135A and H51N mutants the complex with **(I**) and (**II**) inhibitors were either not formed or in a small quantity, respectively. The tautomeric structure [1], (Fig. 4) which is proposed as tetrahedral intermediate [12, 17, 18, 42, 43, 48, 49], could be matched to the observed experimental data in this study only with ambiguity. Indeed, even if we have observed the presence of the minor conformation as a very unstable form for the NS2B:NS3pro with (**I**), we did not observe interaction of G133 in the oxyanion hole, predicted by this structure. The structure [4], (Fig. 4) was earlier proposed but its existence was never proven [18]. It is not clear how the proton from structure [1] could be eliminated leading to structure [4].

Here we propose that NS2B:NS3pro with boronic acid (**I**) can form and exist in a stable form as intermediate [5]. The possibility of the boronic acid inhibitor to form two covalent bond, Ser O^γ^ and His N^ε2^ to boron bonds, in serine protease was postulated in NMR [42, 50–52], and x-ray [16, 17, 49] studies. One way to arrive at this structure is losing a water molecule from intermediate [1, 17, 50].

Despite that both intermediates [5] and [6] (Fig. 4) NS2B:NS3pro with (**1**) and (**II**) inhibitors respectively adopt slightly different strength of LBHB hydrogen bonds observed by NMR between the N^δ1^ proton shared between H51and D75 it cannot account for the large difference in binding affinity of these inhibitors: the former has higher affinity than the latter. One would expect additional stabilization of the intermediate [6] in NS2B:NS3pro with (**II**) inhibitor due to the hydrogen bond of the hemiketal oxygen of the tetrahedral intermediate with an NH of an oxyanion hole amide, G133 and S135, is expected to provide a benefit for the stability of intermediate [6] vs [5] which was not the case. In this study we conclude that presence of LBHB bonds or/and additional hydrogen bonding in the oxyanion hole are necessary but not sufficient factors in stabilization of the intermediate complexes [6] vs to [5]. Other factors could play important roles in the relative stability of these intermediate. One of them is the difference in strength of the formed covalent (H51)N^ε2^ –B bond in tautomer [5] of NS2B:NS3pro with (**I**) vs the hydrogen bond (H51)N^ε2^-H…F in tautomer [6] of NS2B:NS3pro with (**II**). Existence of the latter was proposed in the x-ray study of the structure of chymotrypsin with tfk inhibitor [53]. Noteworthy that in the ^19^F-^1^H Hoesy spectrum (Fig. S11) of the complex NS2B:NS3pro with tfk (**IV**) we have observed an nOe cross peak between the ^19^F nuclei of the CF_3_- group and (H51)H^δ2^ proton supporting the formation of tautomer [6]. Importantly, the total length of the catalytic triad between D75 and the boronic nuclei in [5] is shorter than in [6] between D75 and the hemiketal carbon of inhibitors [5, 6], (Fig. 4). Due to that, a more compact catalytic active site can be formed in the former than in the latter. This means that mimicking of the di-covalent adduct [5] of boronic acid (**I**) could lead to the more potent inhibitor in DENV2 NS2B:NS3 proteases than inhibitor (**II**) mimicking natural substrates. Our finding can play an important role in modelling studies bearing in mind that the computational approach of DENV2 NS2B:NS3 so far reported, has been restricted to covalent serine adducts: for tfk [21], and for boronic acid [29]. The role of compressions of the catalytic triad on the stability of the complex DENV2 NS2B:NS3 proteases as a whole is the subject of our future study.

## Conclusions

In this work, unlinked NS2B:NS3pro and two catalytic NS3pro mutants, S135A and H51N, were studied by NMR spectroscopy with different peptidic inhibitors mimicking the catalytic tetrahedral intermediate. The inhibitors contain the same tetra peptide moiety with different ‘warheads’ (boronic acid (I) and trifluoroketone (II)) on the C-end and bind with different affinity to the DENV2 NS2B:NS3pro.

Our result obtained for the investigated complexes indicates that there are some crucial differences between the conformations adopted in the active site of enzyme. Firstly there is no interaction with the oxyanion hole in the di-covalent adduct [5] formed as intermediate with boronic acid (I), compared to the tetrahedral adduct [6] formed with tfk (II). Secondly, the covalent (H51)N^ε2^ –B bond vs hydrogen bond (H51)N^ε2^-H…F formed for the former vs latter, respectively, the fact that we observe different type of cross-links between protein and ligands we attribute to the plastic topology of the active site of the DENV2 NS2B:NS3pro. We suggest that the boronic ligand (I), poorly resembling a natural substrates carries a promising inhibition property for drug design. The tfk (II) makes good transition-state analogs, binding to the catalytic serine in line with other serine proteases. The finding that the binding site shows preference for different isomers need also be taken into account when designing drugs targeting DENV2 NS2B:NS3pro. This study indicates flexibility, most likely with the different parts being at least partially independent of each other, of the active site of DENV2 NS2B:NS3pro and that further studies are needed to determine if this is indeed the case and if it is the backbone and/or sidechains that are responsible and the magnitude of the indicated flexibility.

Our reported findings will, together with more structural and dynamic measurements on the complex NS3:NS2B:ligand, which is ongoing, facilitate the development of rational structure based inhibitors that can selectively target the NS3 protease of Dengue type II (DENV2) virus.

## Methods

### Protein expression and purification

Reagents were from Sigma (St. Louis, MO, USA) unless otherwise stated. DENV2 NS3pro (1–185; amino acids 1476–1660 of the polyprotein) and NS2B (containing amino acids 1394–1440 of the Dengue 2 polyprotein) constructs were generated as described [10]. H51N and S135A mutations of active site residues were introduced using the QuikChange Lightning kit (Agilent); a double H51N/S135A mutant was generated sequentially. All sequences were confirmed by Sanger sequencing. NS2B and NS3pro were expressed separately in *Escherichia coli* expression strain BL21Star (DE3) (Life Technologies) as described [10]. Proteins were expressed in Terrific Broth medium (MP Biomedicals) for unlabelled protein or in different isotopic labelling combinations in ^1/2^H, ^15^N, ^12/13^C-labelled M9 medium for labelled protein [54]. Chemicals for isotope labelling (ammonium chloride, ^15^N (99%), D-glucose, ^13^C (99%), deuterium oxide) were purchased from Cambridge Isotope Laboratories, Inc.

Incorporation of ^13^C^15^N-histidine (Cambridge Isotope Laboratories) was done as described by [55], NS3pro was grown at 37 °C in Terrific Broth medium until OD_600_ reached 1.3. Cells were then pelleted by centrifugation, washed in PBS and re suspended in pre warmed M9 medium supplemented with 0.2 mM ^13^C^15^N-histidine. After incubation for one additional hour at 230 rpm and 37 °C, expression was induced with IPTG, and cells were harvested by centrifugation after three more hours at 230 rpm and 37 °C.

Purification: NS2B and NS3pro were co-refolded by one-step dialysis overnight at 4 °C in a 2:1 M NS2B:NS3pro ratio to maximize formation of the active complex. The refolding buffer was 25 mM Tris pH 8.5 (pH set at 4 °C), 5% glycerol, 100 mM NaCl. Thrombin (GE Healthcare) and/or TEV protease (produced in house according to [56]) was added to a dialysis cassette (3500 or 7000 MWCO Slide-A-Lyzer, Thermo Fisher Scientific) to cleave off the His tag from NS2B and/or NS3pro. Thrombin could be added directly, and did not lose activity in the high concentration urea solution of the dialysis cassette, while TEV protease was added 1 h after starting the dialysis. After refolding the solution was centrifuged at 50,000×g to remove any precipitate or particles. Refolding yield was determined by measuring protein concentration of the two IMAC pools (NS2B: ε 5500, MW 7.7 kDa; NS3pro: ε 36,400, MW 21.0 kDa) before refolding and comparing that to the protein concentration after refolding and centrifugation (complex: ε 41,940, MW 28.7 kDa), using a Nanodrop 1000 instrument (Thermo Scientific). The complex was then purified on an ÄKTA Explorer (GE Healthcare) by size exclusion on a HiLoad Superdex 200 column (GE Healthcare) in SEC buffer: 50 mM Tris pH 8.5 (4 °C), 5% glycerol, 50 mM NaCl.

### Protease inhibitors

The NS3pro inhibitors Bz-Nle-Lys-Arg-Arg-B(OH)_2_ (**I**) and 2,6-di-fluoro-Bz-Nle-Lys-Arg-Arg-CF3-ketone and Bz-Nle-Lys-Arg-Arg-CF3 (**II-V**) used in this study were synthesized according to the reaction schemes published in the original paper [44].

### Biochemical assay

Activity assays were carried out on a 96-well plate (white Cliniplate, Thermo Fisher Scientific Oy, Vantaa, Finland) in 50 mM HEPES, pH 7.4, 150 mM NaCl, 10% ethylene glycol, 0.05% BSA, 0.0016% Brij-58 with 80 nM enzyme using 20 μM Bz-nle-Lys-Arg-Arg-AMC (Bachem, Bubendorf, Switzerland) as substrate. Reagents were from Sigma (St. Louis, MO, USA) unless otherwise stated.

5 μl of 200 μM substrate in buffer and 2.5 μl of compound in DMSO or DMSO control were added to the plate. 42.5 μl of 94 μM enzyme in buffer was added to start the reaction. Fluorescence was read every 30 s for 30 min at 390 nm excitation and 460 nm emission in a Fluorskan Ascent plate reader (Thermo Fisher Scientific Oy, Vantaa, Finland). Rates were fitted in the Ascent software and exported as Excel files. The rates were imported into GraphPad Prism (GraphPad Software Inc., La Jolla, CA, USA) and fitted to the standard three parameter IC_50_ equation.

### NMR samples preparation

The NS2B:NS3pro complex was concentrated in disposable centrifugal concentrators (e.g. Amicon Ultra centrifugal filter units) with a molecular weight cut-off of 10 kDa. The complex was stable during concentration and no leakage of NS2B occurred. Buffer was exchanged using gravity flow desalting columns (GE Healthcare). The NMR buffer contained 20 mM deuterated MES, 100 mM NaCl, 5 mM CaCl_2_, 0.02% NaN_3_, at pH 6.5. The buffer-exchanged protein was concentrated to at least 0.3 mM. To obtain spectra in D_2_O buffer protein complex with inhibitor was lyophilized and dissolved adding only D_2_O water.

### NMR spectroscopy

NMR experiments were acquired on Bruker Avance III spectrometers operating at 14.1 and 16.4 T at a temperature of 298 K. 2D ^1^H-^15^N TROSY -transverse relaxation optimized spectroscopy (TROSY) was used [57–59]. ^19^F experiments were acquired on a Bruker Avance III 16.4 T equipped with a QCIF CryoProbe, and internally referenced to the trifluoromethyl acetate counter ion (− 76.55 ppm). For solvent isotope shift measurements the buffer was exchanged from 90% H_2_O to 90% D_2_O using centrifugation filters by following dilution of the sample till starting concentration. The consistency of the procedure has been tested through comparison of the ^1^H-^15^N spectra before and after buffer exchange back to starting condition. There was no major structural perturbations resulted from exchange into deuterated buffer conditions. Protein detected experiments with ligand bound used a chemical shift perturbation (CSP) analysis was performed manually in CcpNmr Analysis 2.2.2 [60]. CSP was defined as the distance between two cross peaks in Hz, obtained as the square root of the sum of quadratic using DANGLE [61].

### Protein assignment

Backbone resonance assignment of the NS2B:NS3pro with tetra peptide boronic acid (**I**) was performed as described previously by us with depositing to the BioMagResBank with accession code **26996** [10, 62], and compared with published earlier for the same inhibitor complex but with the linked construct Dengue I NS2B:NS3pro (BioMagResBank with accession code 19305). Backbone resonance assignment have been done in the same manner for the complex NS2B:NS3pro with the tetra peptide tfk ligand (**II**) as well and will be published elsewhere. Partial assignments of the ^1^H and ^15^N resonances presented in this publication of the apo form NS2B:NS3pro and its mutants in complex with inhibitors observed to the uncrowded area of the ^1^H-^15^N spectra are made by the extrapolation of corresponding cross peaks to the closest one known amino acids using CCPN program tool. The assignment both aromatic and imide ^1^H and ^15^N resonances of the three histidines (His) presented in sequence of the NS3pro was subsequently confirmed by the comparison between the ^1^H-^15^N HSQC spectra of the enzymes: the complex uniformly labelled ^2^H^13^C^15^N-NS3pro and unlabeled NS2B versus specifically ^15^N^13^C His labelled NS3pro on the unlabelled background and unlabeled NS2B. Both samples are in complex with the boronic acid type inhibitor, (**I**), inhibitor (see additional Supporting Information).

## Supplementary information

**Additional File 1: Fig. S1.** Superposition of ^19^F spectra of (IV) with adding NS2B:NS3pro. **Fig. S2**: Extensions of ^19^F spectra of (II). **Fig. S3**: Superposition of the ^1^H-^15^N TROSY spectrum and 2D plane ^1^H-^15^N of 3D experiment of (a) ^15^N^13^C^2^H labeled NS2B:NS3pro with (I) and of (b) ^15^N^13^C^2^H labeled NS3pro unlabeled NS2B with (IV). **Fig. S4**: CSP between the amide resonances of NS3pro of the complexes with (I) and (II). **Fig. S5**: Superposition of the ^1^H-^15^N TROSY spectra of the NS2B:NS3pro with selectively labelled ^15^N-His’s of apo , and in complex with(I) and with (II). **Fig. S6**: The superposition of ^19^F spectra of the complex NS2B:NS3pro with (II) at different pH. **Fig. S7**: ^1^H-^15^N TROSY spectrum of the apo forms of the ^15^N^13^C labeled S135A-mutant of NS2B:NS3pro with spectrum of the NS2B:NS3pro apo . **Fig. S**8a: Superposition of the 1H-15 N TROSY spectrum of the apo forms of the ^15^N^13^C labeled S135A-mutant of NS2B:NS3pro and in complex with (I) and (II). Fig. **S8**b: Superposition of^19^F spectra of (II). **Fig. S9**a: ^1^H-^15^N TROSY spectrum of the apo forms of the ^15^N^13^C labeled H51N-mutant of NS2B:NS3pro overlies with spectrum of the wild type NS2B:NS3pro apo. Fig. **S9**b: Superposition of the ^1^H-^15^N TROSY spectra of the apo forms of the ^15^N^13^C labeled S135A vs H51N-mutant of NS2B:NS3pro. Fig. **S10**a: Superposition of ^19^F spectra of (II). **Fig. S10**b: Superposition of the ^1^H-^15^N TROSY spectra of the ^15^N^13^C labeled H51N-mutant with (II) and following addition of (I). **Fig. S10**c: Superposition of the ^1^H-^15^N TROSY spectra of mixture of the ^15^N^13^C labelled H51N-mutant with (II) and following addition of (I) vs apo form. **Fig. S11**: ^19^F -^1^H Hoesy spectrum of the complex NS2B:NS3pro with(IV).

## Data Availability

The datasets generated during this study are available from the corresponding author on reasonable request.

## Abbreviations

DENV2: Dengue serotype II virus
NMR: Nuclear magnetic resonance
Tfk: Trifluoromethyl ketone
LBHB: Low barrier hydrogen bond
WNV: Western Nile virus
YFV: Yellow fever virus
CSP: Chemical shift perturbation
HCV: Hepatite C virus
Tris: Tris(hydroxymethyl)aminomethane
MES: 2-(N-morpholino) ethanesulfonic acid
^1^H-^15^N TROSY: ^1^H-^15^N transverse relaxation-optimized spectroscopy
