# Supplementary material for: Probing contacts of inhibitor locked in transition states in the catalytic triad of DENV2 type serine protease and its mutants by 1H, 19F and 15 N NMR spectroscopy
Source: BMC Mol Cell Biol. 2020 May 25;21:38. doi: 10.1186/s12860-020-00283-0 (PMC7249419; doi:10.1186/s12860-020-00283-0)
Supplement: Supplementary file 1 — Additional File 1: Fig. S1. Superposition of 19F spectra of (IV) with adding NS2B:NS3pro. Fig. S2: Extensions of 19F spectra of (II). Fig. S3: Superposition of the 1H-15N TROSY spectrum and 2D plane 1H-15N of 3D experiment of (a) 15N13C2H labeled NS2B:NS3pro with (I) and of (b) 15N13C2H labeled NS3pro unlabeled NS2B with (IV). Fig. S4: CSP between the amide resonances of NS3pro of the complexes with (I) and (II). Fig. S5: Superposition of the 1H-15N TROSY spectra of the NS2B:NS3pro with selectively labelled 15N-His’s of apo , and in complex with(I) and with (II). Fig. S6: The superposition of 19F spectra of the complex NS2B:NS3pro with (II) at different pH. Fig. S7: 1H-15N TROSY spectrum of the apo forms of the 15N13C labeled S135A-mutant of NS2B:NS3pro with spectrum of the NS2B:NS3pro apo . Fig. S8a: Superposition of the 1H-15 N TROSY spectrum of the apo forms of the 15N13C labeled S135A-mutant of NS2B:NS3pro and in complex with (I) and (II). Fig. S8b: Superposition of19F spectra of (II). Fig. S9a: 1H-15N TROSY spectrum of the apo forms of the 15N13C labeled H51N-mutant of NS2B:NS3pro overlies with spectrum of the wild type NS2B:NS3pro apo. Fig. S9b: Superposition of the 1H-15N TROSY spectra of the apo forms of the 15N13C labeled S135A vs H51N-mutant of NS2B:NS3pro. Fig. S10a: Superposition of 19F spectra of (II). Fig. S10b: Superposition of the 1H-15N TROSY spectra of the 15N13C labeled H51N-mutant with (II) and following addition of (I). Fig. S10c: Superposition of the 1H-15N TROSY spectra of mixture of the 15N13C labelled H51N-mutant with (II) and following addition of (I) vs apo form. Fig. S11: 19F -1H Hoesy spectrum of the complex NS2B:NS3pro with(IV). [file 12860_2020_283_MOESM1_ESM.pdf]

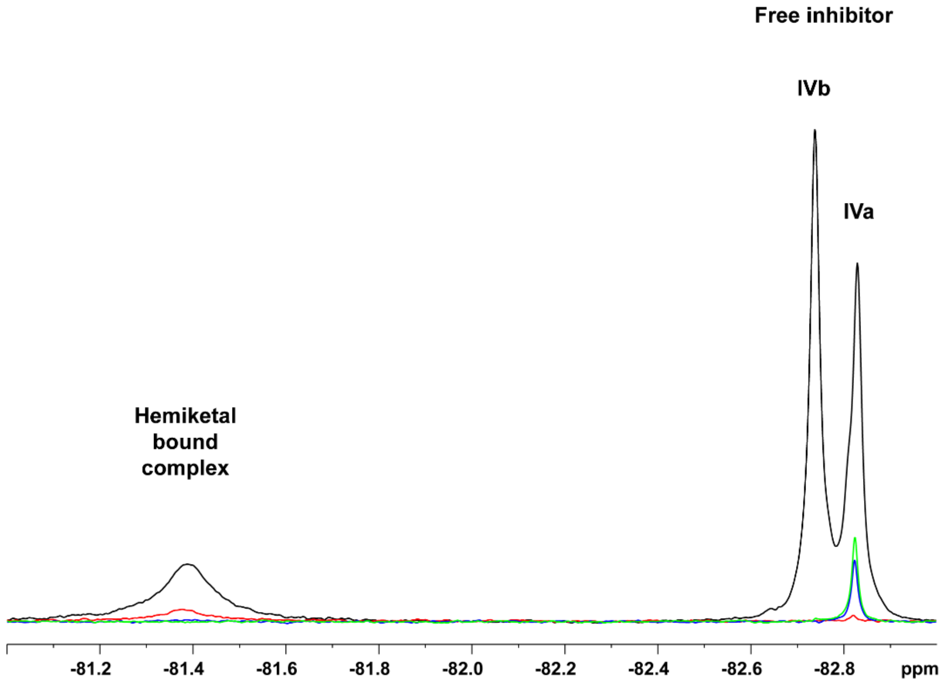

Fig S1. Superposition of the ^{19}F spectra pro between -80.0 and -83.0 ppm: (black) of the tfk (**IV**) with adding NS2B:NS3pro obtained (in ratio 1.6:0.6mM correspondingly); (red) after exchange buffer and "wash out" free isomers, (concentration of the complex 0.28mM); (blue) following addition of boronic (**I**) (in ratio 0.28:0.625mM); (green) after one week incubation 25°C are shown.

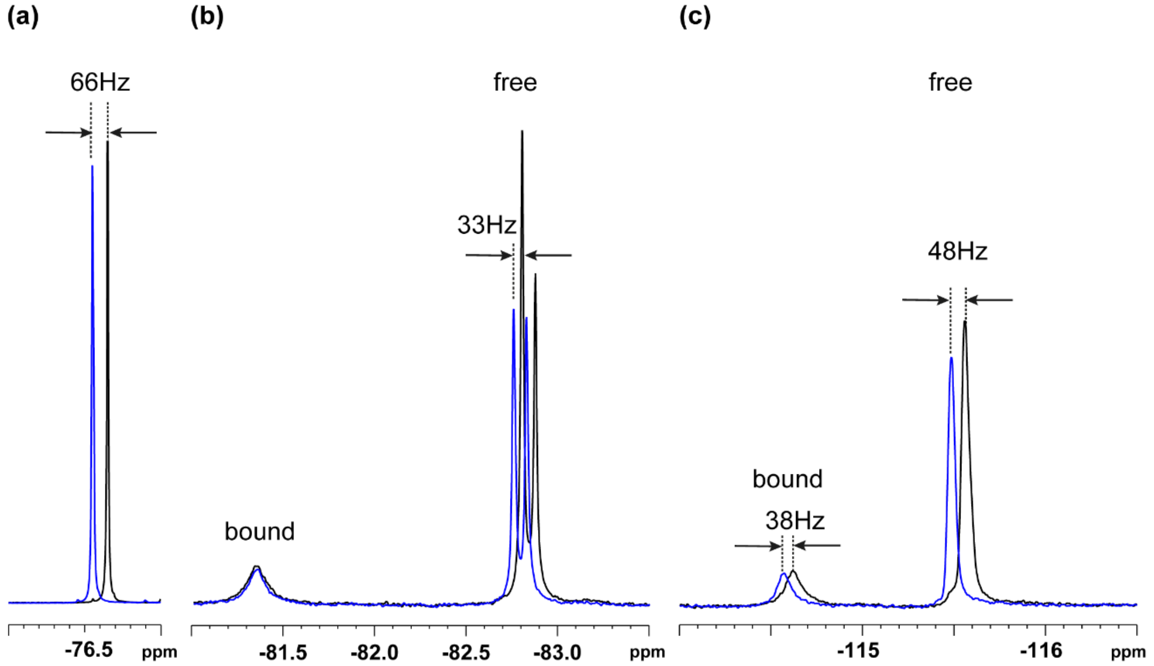

Fig S2 Extensions of the ^{19}F spectra between: (a) -76.0 and -77.0ppm showing the reference signal TFA; (b) and (c) with resonances of bound and free inhibitor, 2, 6-di-fluoro-Bz-Nle-Lys-Arg-Arg-CF_3-ketone (**II**) in regions -81.0 till 83.5ppm and -114 till -116.5 showing ^{19}F of CF_3-ketone and 2, 6-di-fluoro-Bz groups, respectively. The spectrum presented with blue line was obtained in 90%H2O/10%D2O buffer solution, with black line: in 100% D_2O buffer solution.

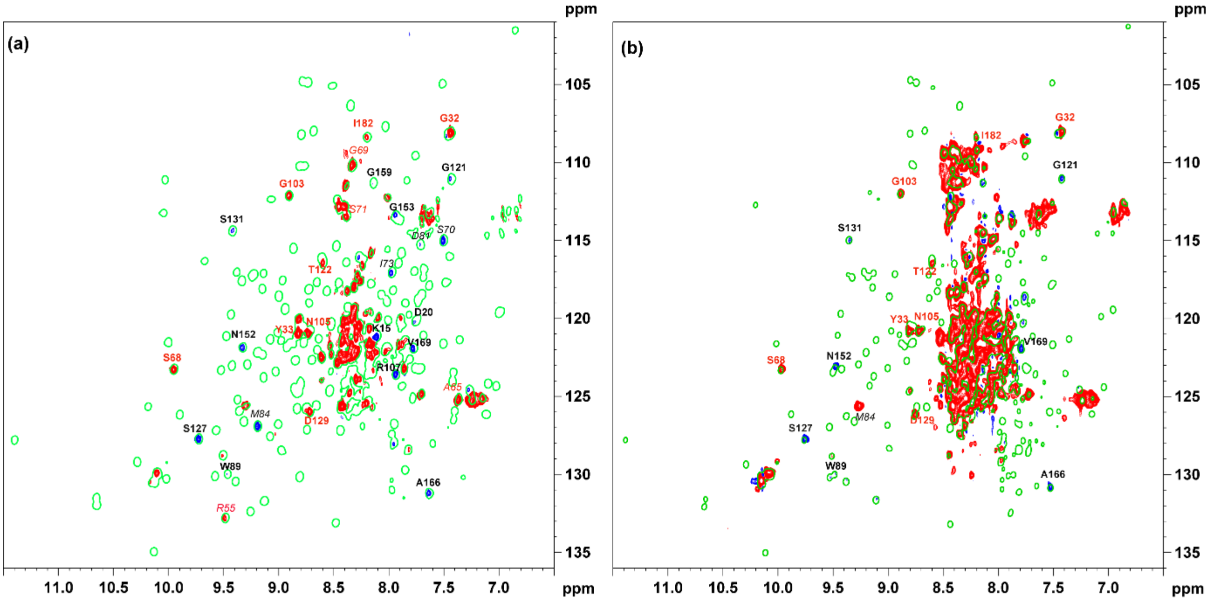

Fig S3 Superposition of the ^{1}H-^{15}N TROSY spectrum (green) and 2D plane ^{1}H-^{15}N of 3D experiment of (a) ^{15}N^{13}C^{2}H labeled NS2B:NS3pro with boronic acid (**I**) inhibitor and of (b) ^{15}N^{13}C^{2}H labeled NS3pro unlabeled NS2B with tfk (**IV**) inhibitor where cross peaks belonging to the nOe contacts between NH protons and bound water protons shown by blue, but NH protons in exchange with bulk water shown by red.

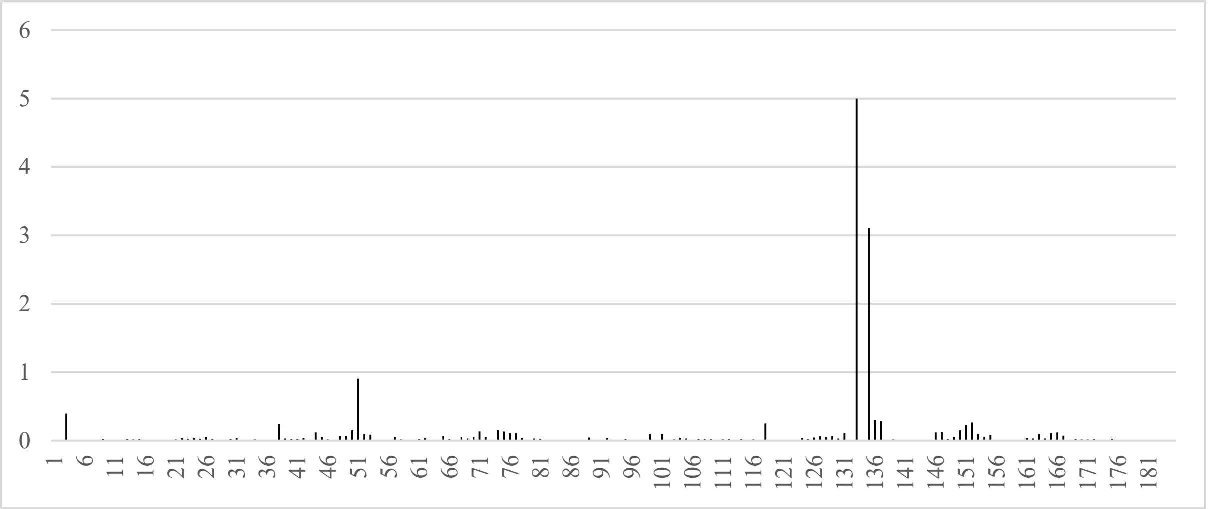

Fig S4 CSP between the amide resonances of NS3pro of the complexes with boronic acid (I) and tfk (II).

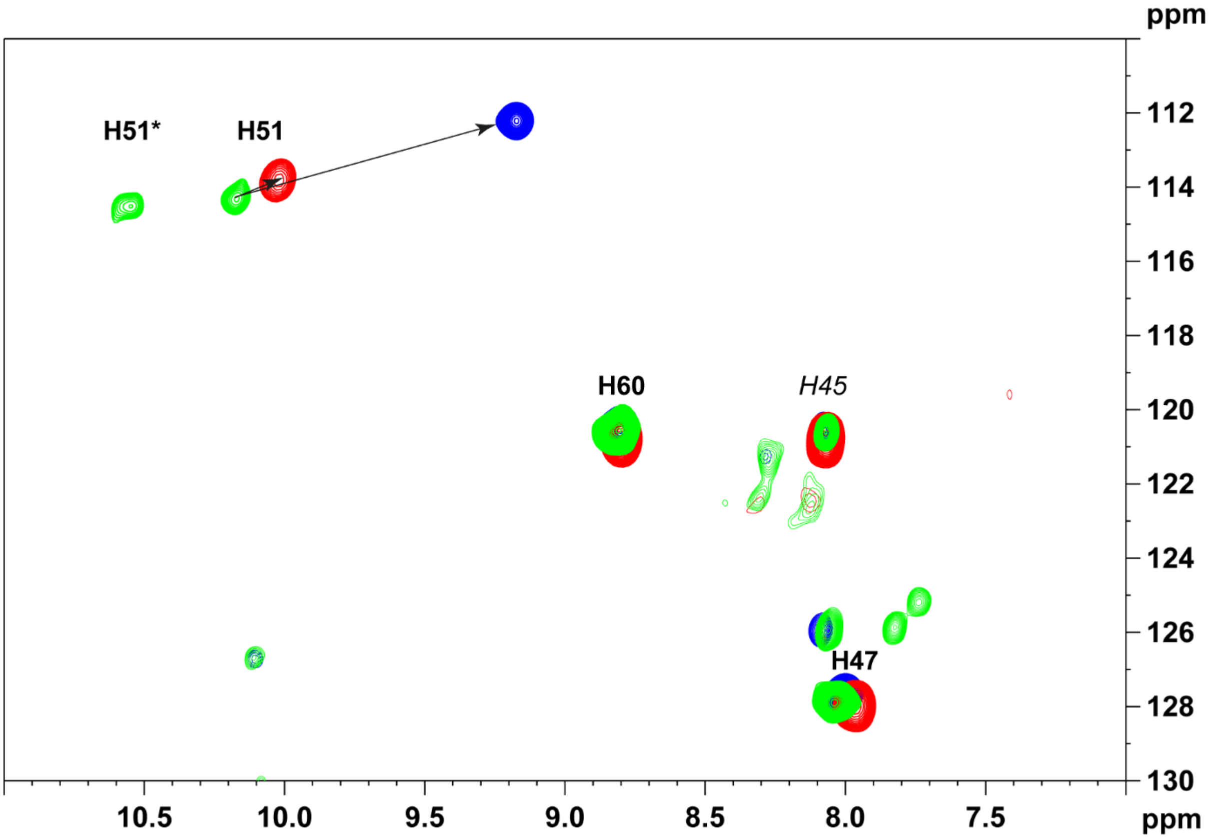

Fig S5 Superposition of the ^{1}H-^{15}N TROSY spectra of the NS2B:NS3pro with selectively labelled ^{15}N-His's of apo (green), and in complex with boronic (**I**) (red) and with tfk (**II**) (blue) inhibitors. The cross peaks belonging to NS3pro, H51, H60, H47 are shown in bold. The cross peak belonging to NS2B, H45 is shown in italic. By H51* is labelled second amide H51cross peak belonging to another conformation. The ratio between these two cross peaks are dependent on sample preparation

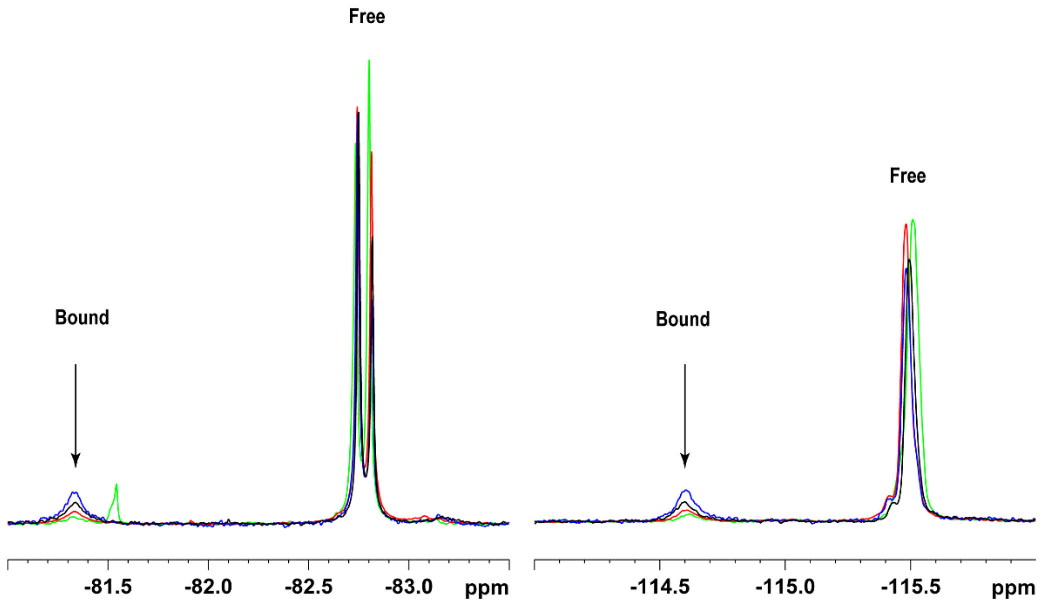

Fig S6 The superposition of the extended ^{19}F spectra of the complex NS2B:NS3pro with tfk (**II**) between: -81.0 and -83.5.0ppm and -114.0 till -116.0ppm showing ^{19}F resonances of the CF_3-ketone and 2, 6-di-fluoro-Bz groups of tfk (**II**) inhibitor correspondingly depending from pH of buffer: 5.5 (black), 6.0 (blue), 7.2 (red) and 8.5 (green).

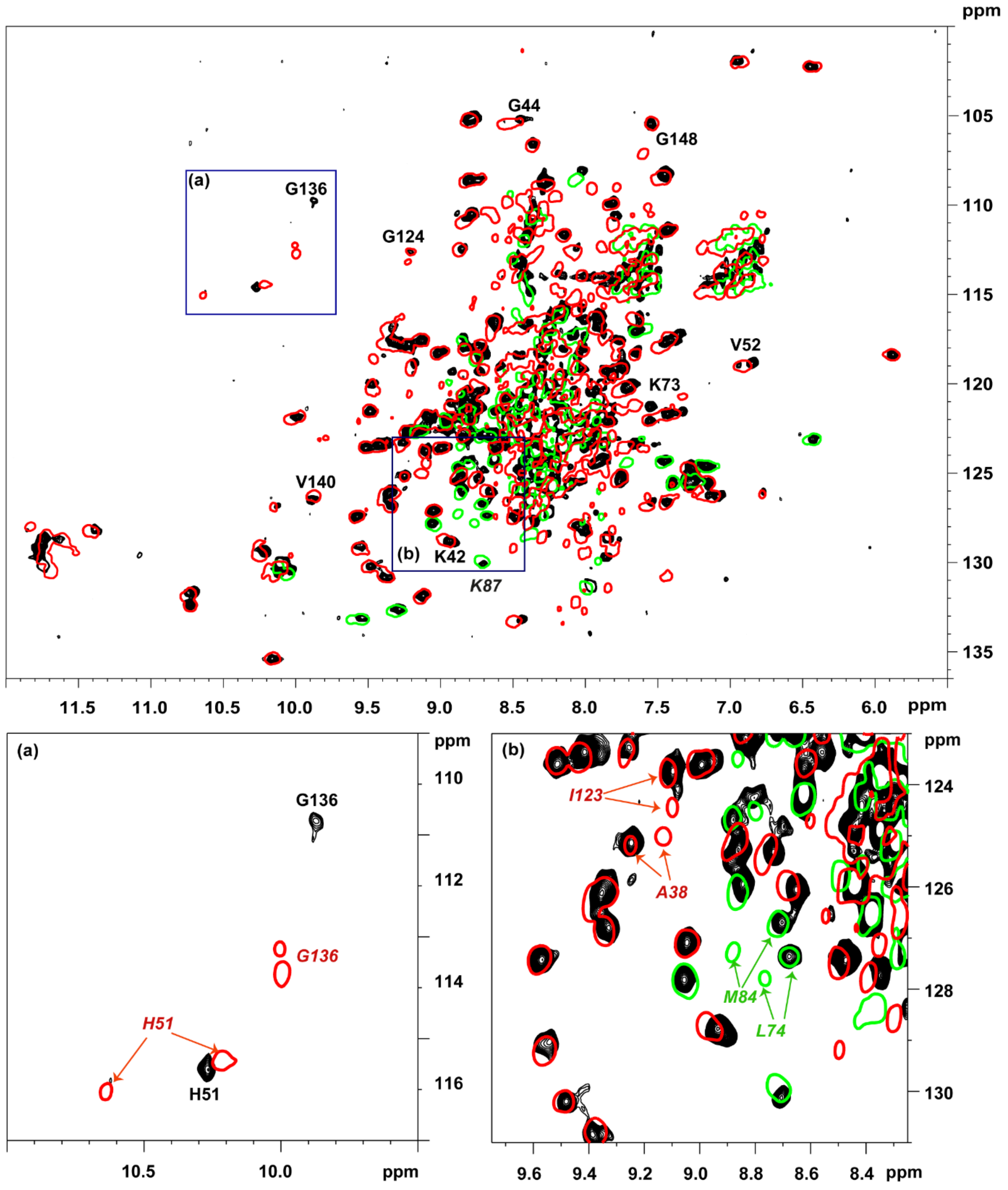

Fig S7 ^{1}H-^{15}N TROSY spectrum of the *apo* forms of the $^{15}N^{13}C$ labeled S135A-mutant of NS2B:NS3pro (black) overlies with spectrum of the NS2B:NS3pro apo: (green) $^{15}N^{13}C^{2}H$ labeled NS2B with unlabeled NS3 and (red) $^{15}N^{13}C^{2}H$ labeled NS3 with –unlabeled NS2B. The spectra of both complexes are very similar. The largest shifts were observed for the following residues: K42, G44, H51, V52, G136, V140 belonging to the NS3 and K87 belonging to the NS2B (marked with bold black or italic bold black, respectively, according to the residue number in sequence). Panels (a) and (b) exhibit extended part of spectrum showing the cross peaks of amino acids observed as doubled in wild NS2B:NS3pro apo and single in S135A-mutant.

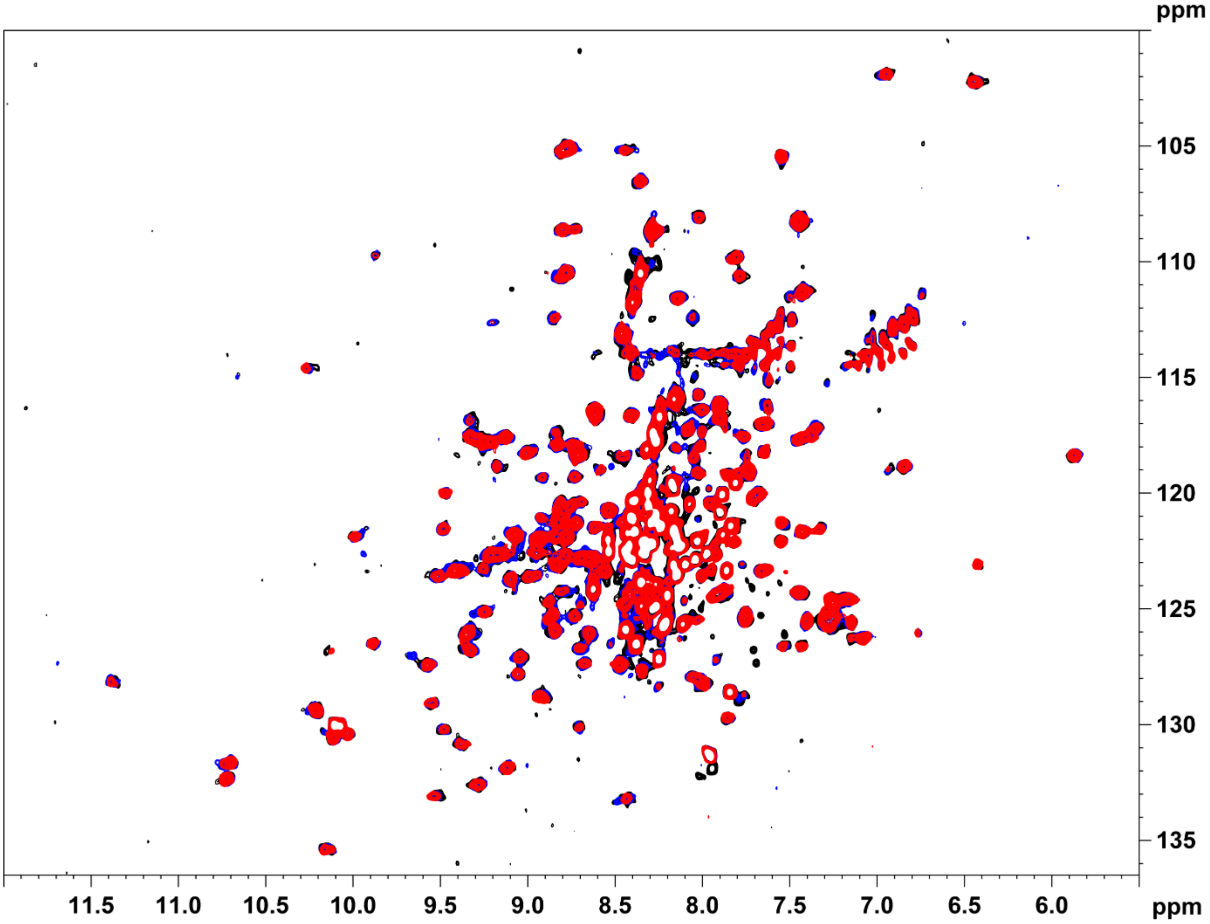

Fig S8 (a). Superposition of the ^{1}H-^{15}N TROSY spectrum of the *apo* forms of the $^{15}N^{13}C$ labeled S135A-mutant of NS2B:NS3pro (black) and in complex with boronic (**I**) (blue) and with tfk (**II**) (red) inhibitors. The observed spectra are almost identical indicating the absent of interaction between apo form of mutant and inhibitors.

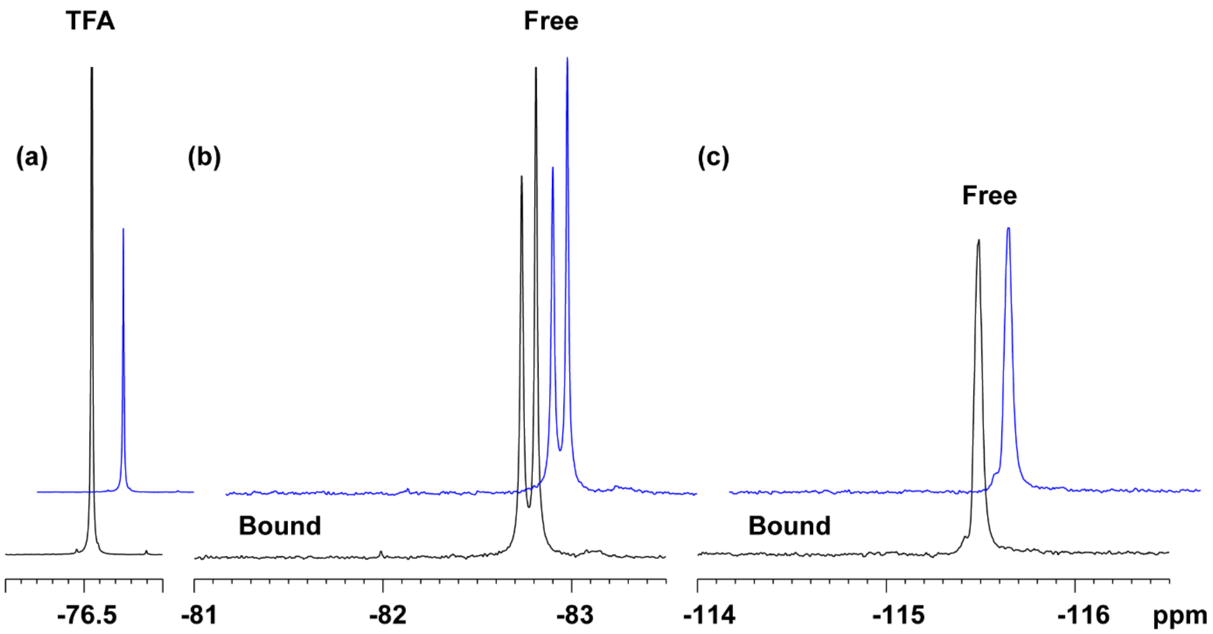

Fig S8 (b). Superposition of the extended ^{19}F spectra between: (a) -76.0 and -77.0ppm showing the reference signal TFA, and in regions -81.0 till -83.5ppm (b) and -114 till -116.5ppm (c) which are the place of locations of the ^{19}F resonances of the CF_3-ketone and 2, 6-di-fluoro-Bz groups of the 2, 6-di-fluoro-Bz-Nle-Lys-Arg-Arg-CF_3-ketone (**II**) inhibitor correspondingly. The spectrum presented by black line belongs to the complex of the *apo* forms of the S135A-mutant with tfk (**II**) inhibitor. The spectrum presented by blue line belong to the complex of the S135A-mutant with tfk (**II**) inhibitor with following adding of boronic (**I**) inhibitor. No bound complex formation was observed.

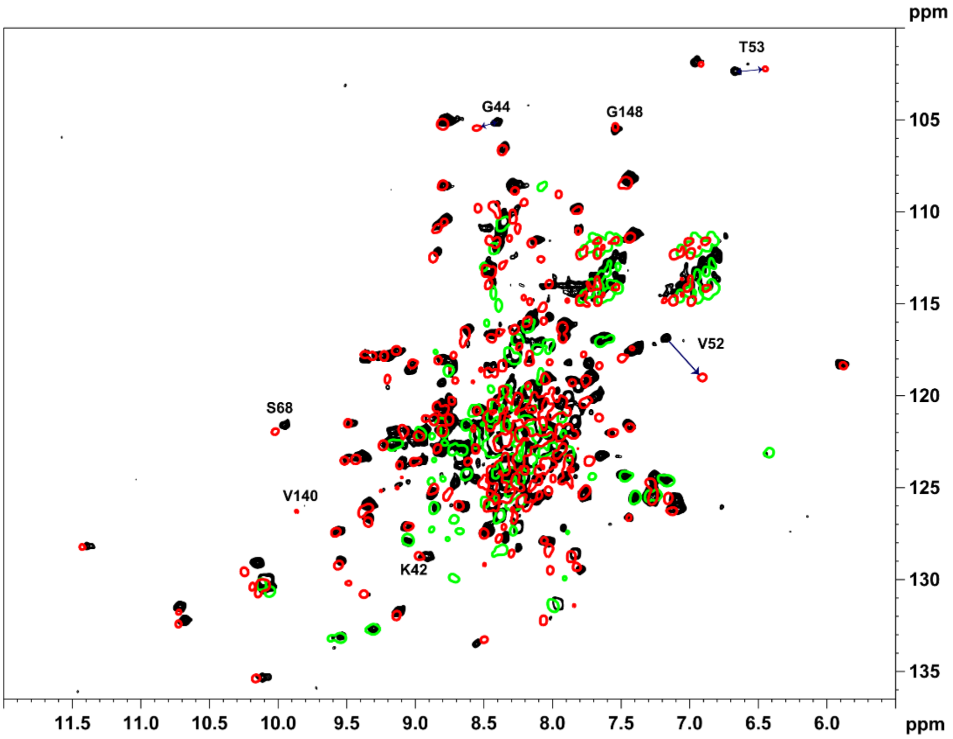

Fig S9(a) ^{1}H-^{15}N TROSY spectrum of the *apo* forms of the ^{15}N^{13}C labeled H51N-mutant of NS2B:NS3pro (black) overlies with spectrum of the wild type NS2B:NS3pro apo: (green) ^{15}N^{13}C^{2}H labeled NS2B with unlabeled NS3 and (red) ^{15}N^{13}C^{2}H labeled NS3 with unlabeled NS2B. The spectra of both complexes are very similar. The largest shifts were observed for the following residues: V**52**, T**53**, R**54,** G**55**, and G**44** 84Hz belonging to the NS3pro (marked with bold according to the residue number in sequence).

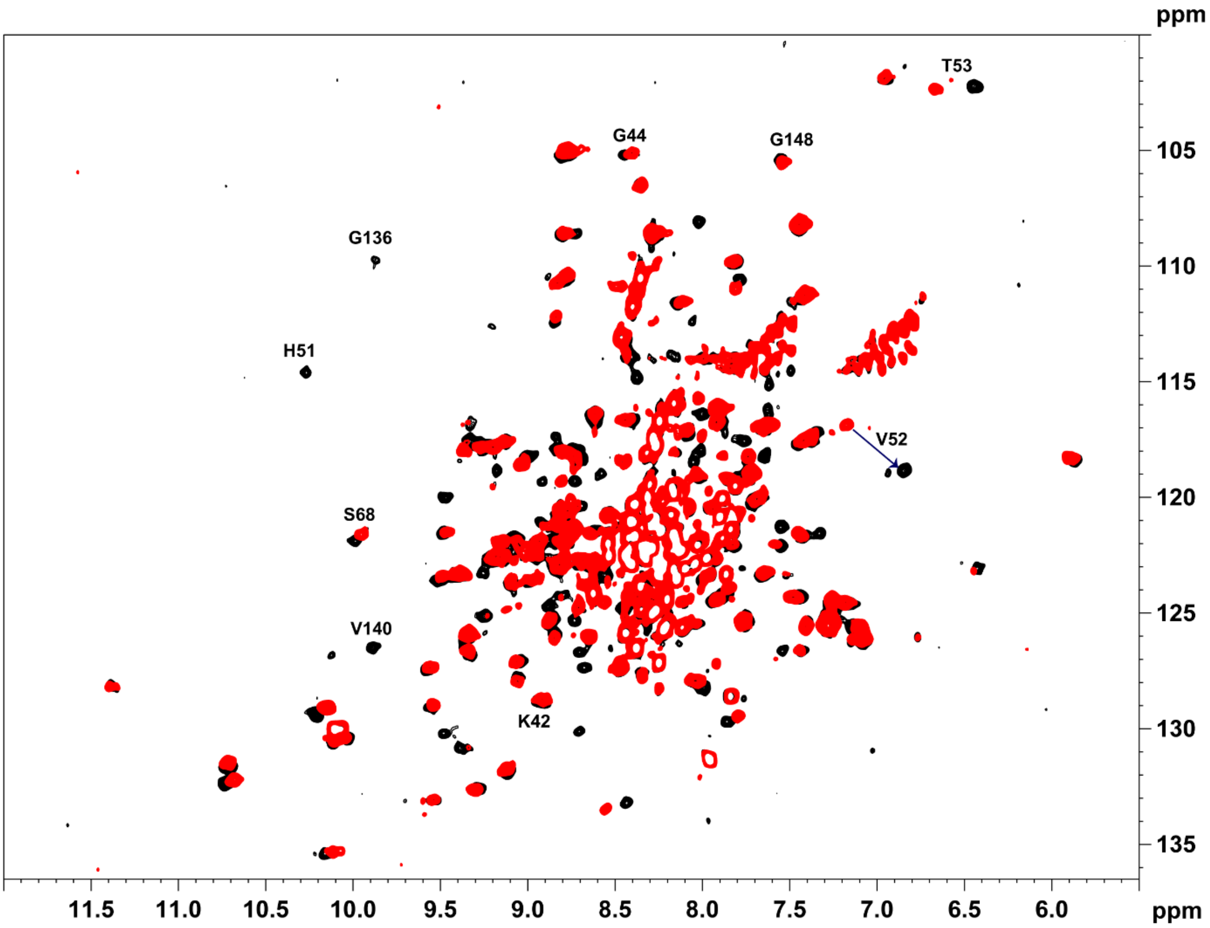

Fig S9(b) The superposition of the ^{1}H-^{15}N TROSY spectra of the *apo* forms of the $^{15}N^{13}C$ labeled (black) S135A vs (red) H51N-mutant of NS2B:NS3pro. The spectra of both complexes are very similar. The largest shifts were observed for the following residues: V**52**, T**53** of NS3pro (marked with bold according to the residue number in sequence). Noteworthy some cross peaks observed in spectrum of S135A are broadened below detection in spectrum H51N: as example G136, H51, V140.

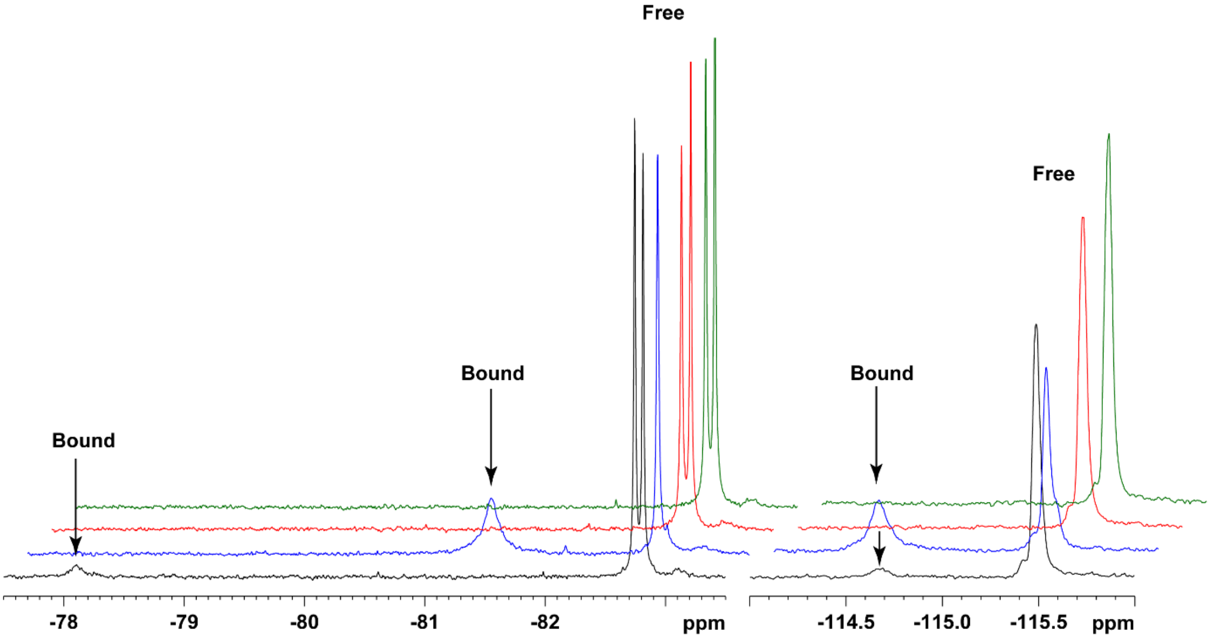

Fig S10 (a). Superposition of the extended ^{19}F spectra between: -77.5 ÷ -83.5ppm (CF_3-ketone) and -114.0 ÷ -116.5ppm (2, 6-di-fluoro-Bz groups) of the tfk (**II**) inhibitor. The following spectra of the tfk (**II**) inhibitor with H51N-mutant (black), wild type of NS2B:NS3pro (blue) and S135A-mutant (red) are presented with following adding of the boronic (**I**) inhibitor in green.

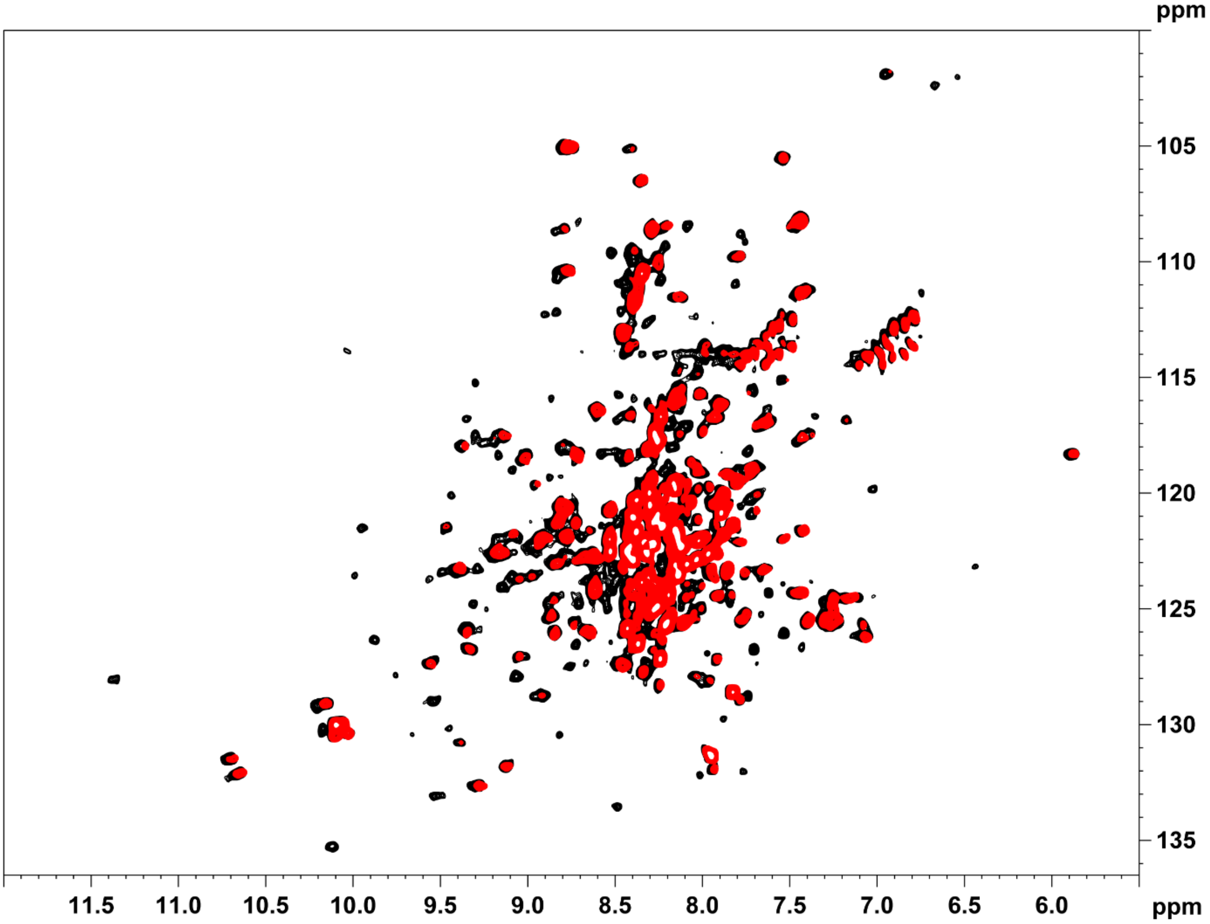

Fig S10 (b). Superposition of the ^{1}H-^{15}N TROSY spectra of the $^{15}N^{13}C$ labeled H51N-mutant with tfk (**II**) inhibitor (black) and with following adding of boronic (**I**) inhibitor (red). A small fraction of complex of H51N-mutant with tfk (**II**) (black) is clearly seen through appearing of the cross peaks with weak intensities.

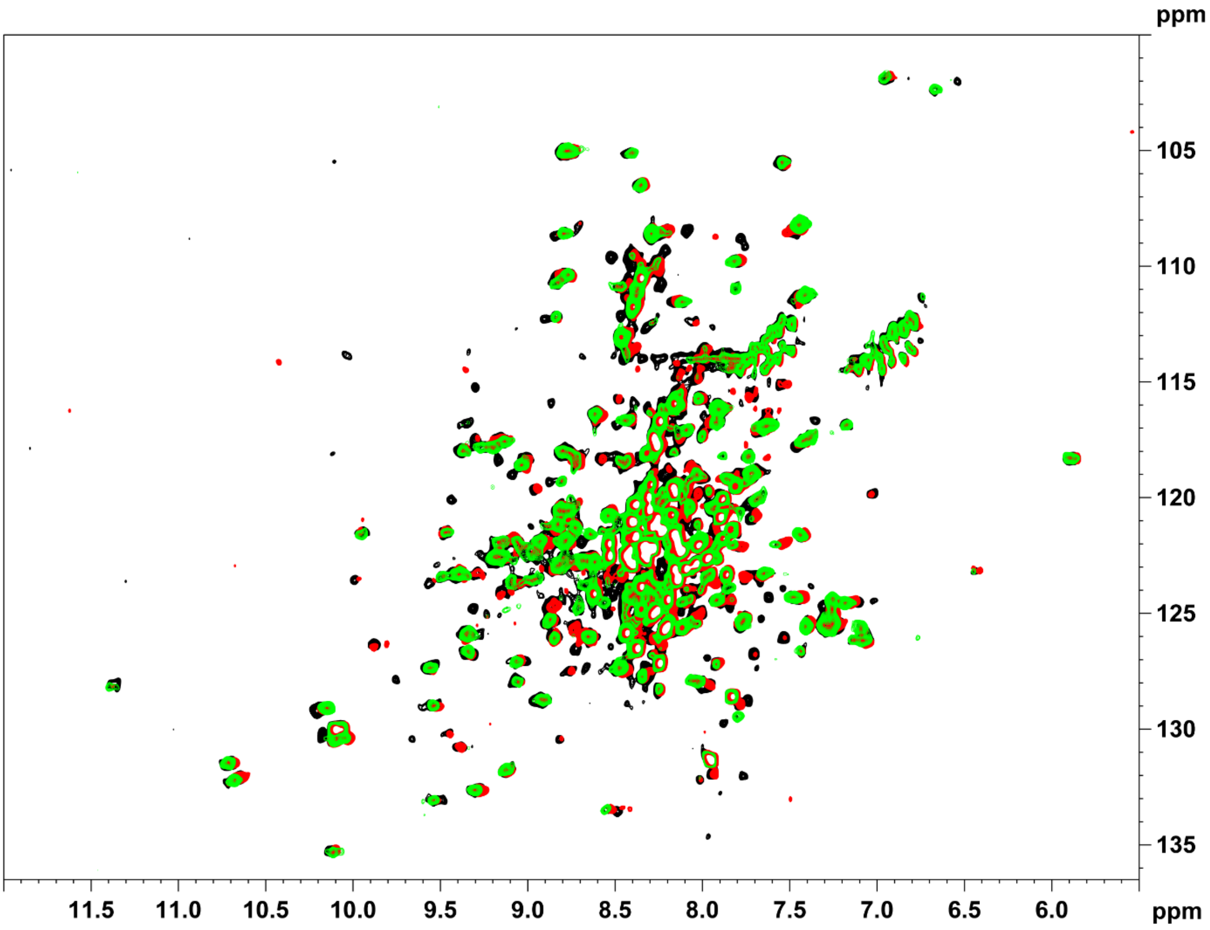

Fig S10 (c). Superposition of the ^{1}H-^{15}N TROSY spectra of mixture of the $^{15}N^{13}C$ labelled H51N-mutant with tfk (**II**) inhibitor (black) and with following adding of boronic (**I**) inhibitor (red) vs its apo form (green).

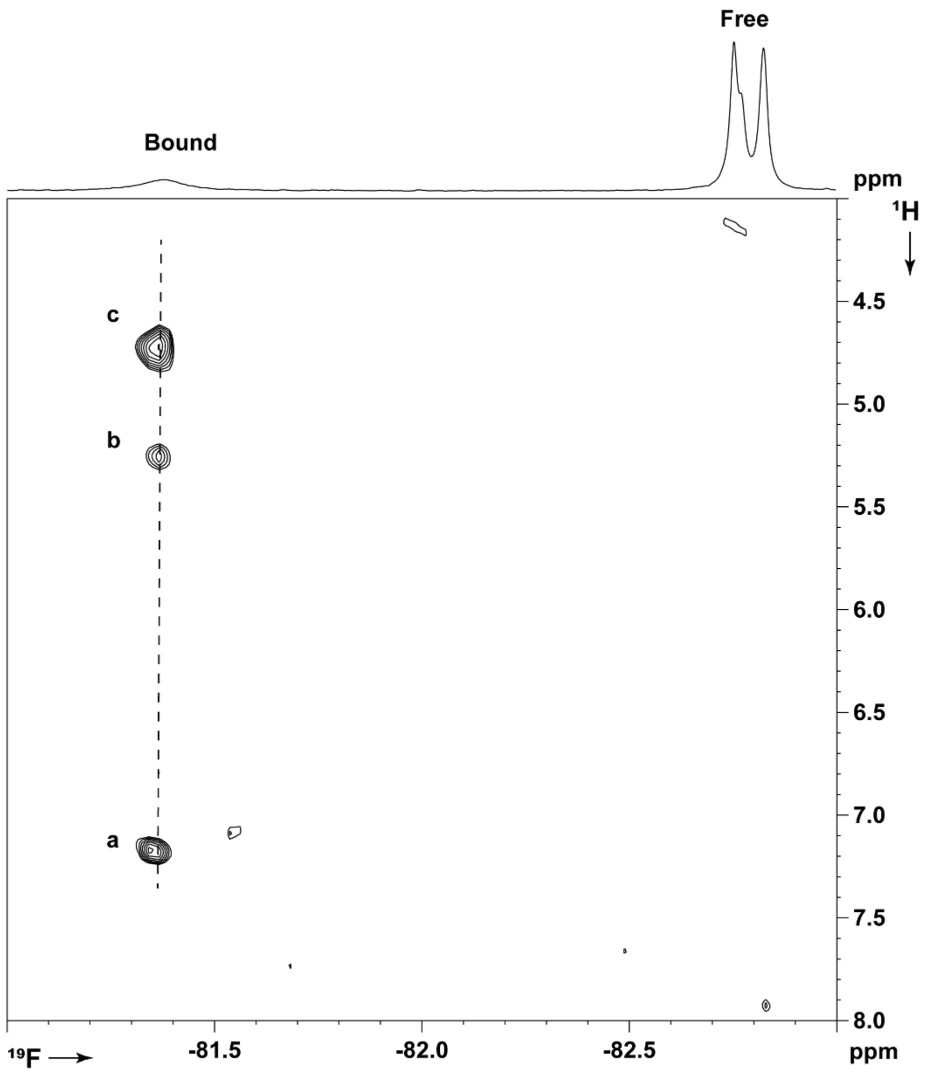

Fig S11 ^{19}F -^{1}H Hoesy spectrum of the complex NS2B:NS3pro with tfk (**IV**) between: -81.0 and -83.0ppm showing nOe contacts between ^{19}F resonances of the CF_3-ketone group with protein protons in bound state: (a) with aromatic $H^{\delta 2}$ of H51.
